# ADAMTS Sol narae cleaves extracellular Wingless to generate a novel active form that regulates cell proliferation in *Drosophila*

**DOI:** 10.1038/s41419-019-1794-8

**Published:** 2019-07-22

**Authors:** Jong-Hoon Won, Go-Woon Kim, Ja-Young Kim, Dong-Gyu Cho, Buki Kwon, Young-Kyung Bae, Kyung-Ok Cho

**Affiliations:** 1 Department of Biological Sciences, Korea Advanced Institute of Science and Technology, 291 Daehak-ro, Yuseong-gu, Daejeon, Korea; 2 Center for Bioanalysis, Korea Research Institute of Standards and Science, 267 Gajung-ro, Yuseung-gu, Daejeon, Korea

**Keywords:** Extracellular signalling molecules, Cell proliferation

## Abstract

Wnt/ Wingless (Wg) is essential for embryonic development and adult homeostasis in all metazoans, but the mechanisms by which secreted Wnt/Wg is processed remain largely unknown. A *Drosophila* Sol narae (Sona) is a member of **A** **D**isintegrin **A**nd **M**etalloprotease with **T**hrombo**S**pondin motif (ADAMTS) family, and positively regulates Wg signaling by promoting Wg secretion. Here we report that Sona and Wg are secreted by both conventional Golgi and exosomal transports, and Sona cleaves extracellular Wg at the two specific sites, leading to the generation of N-terminal domain (NTD) and C-terminal domain (CTD) fragments. The cleaved forms of extracellular Wg were detected in the extracellular region of fly wing discs, and its level was substantially reduced in *sona* mutants. Transient overexpression of Wg-CTD increased wing size while prolonged overexpression caused lethality and developmental defects. In contrast, Wg-NTD did not induce any phenotype. Moreover, the wing defects and lethality induced by *sona* RNAi were considerably rescued by Wg-CTD, indicating that a main function of extracellular Sona is the generation of Wg-CTD. Wg-CTD stabilized cytoplasmic Armadillo (Arm) and had genetic interactions with components of canonical Wg signaling. Wg-CTD also induced Wg downstream targets such as Distal-less (Dll) and Vestigial (Vg). Most importantly, Cyclin D (Cyc D) was induced by Wg-CTD but not by full-length Wg. Because Sona also induces Cyc D in a cell non-autonomous manner, Wg-CTD generated by Sona in the extracellular region activates a subset of Wg signaling whose major function is the regulation of cell proliferation.

## Introduction

Cellular communication via components in the extracellular matrix (ECM) is essential for cell survival and proliferation as well as differentiation. Extracellular proteases play important roles in regulating activity, localization and stability of the ECM proteins^1–3^. Despite the importance of these proteases, their specific functions are still largely unexplored. ADAMTS family contains extracellular proteases that are present only in metazoans^4,5^. Six and nineteen members have so far been identified in flies and mammals, respectively^6,7^. Mammalian ADAMTSs are involved in cell proliferation, angiogenesis and organogenesis, so their malfunctions result in various diseases such as cancer, arthritis, and arteriosclerosis^7–9^. An ADAMTS Sol narae (Sona) is essential for fly development^10^. Loss of *sona* decreases the level of extracellular Wg, and *sona* exhibits positive genetic interaction with *wntless* (*wls*) that encodes a cargo protein for Wg^10–12^. Therefore, intracellular Sona seems to cooperate with Wls in Wg secretion^10^. We recently reported a new function of extracellular Sona in cell survival and cell proliferation^13^. *sona* has genetic interactions with cell death-related genes such as *Death-associated inhibitor of apoptosis* (*Diap1*) and *reaper*. Interestingly, Sona upregulates Cyclin D (Cyc D) in a cell non-autonomous manner, and increases tissue size. Cyc D is a G1 Cyclin to initiate the cell cycle by responding to the mitogen signals^14^. Therefore, it is possible that extracellular Sona generates a yet unidentified signaling molecule that induces Cyc D in the signal-receiving cells.

Wnt family is essential for animal development, and has been extensively studied since a mutant of fly Wg, the homolog of vertebrate Wnt1, was described a century ago^15–17^. Wnt is secreted by both conventional Golgi-mediated transport and exosomal secretion pathway^18–20^. Interaction between Wnt and Frizzled (Fz) receptors initiates a cascade of intracellular responses in the responding cells that lead to downstream gene expression^21,22^. In flies, Wg is involved in cell proliferation, differentiation, and survival by inducing Wg effector components including Vestigial (Vg), Distal-less (Dll) and Senseless (Sens)^23–26^. In mammals, Wnt signaling promotes cell proliferation by transcriptional activation of multiple target genes such as c-Myc and Cyc D^27–29^ and its malfunction leads to various diseases such as cancer, neurodegenerative diseases, inflammatory disease, and diabetes^30–32^.

We asked the role of extracellular Sona in this study and found that Sona generates NTD and CTD fragments of Wg by cleaving extracellular Wg. The Wg-CTD fragment was similar to full-length Wg in activating canonical Wg signaling but was dissimilar to full-length Wg in Cyc D induction, lack of Sens induction, and protein instability. Thus, one of the main functions of Sona is to generate Wg-CTD that carries out subsets of Wg signaling.

## Results

### Sona and Wg are secreted by both conventional Golgi and exosomal transports

**Fig. 1:**
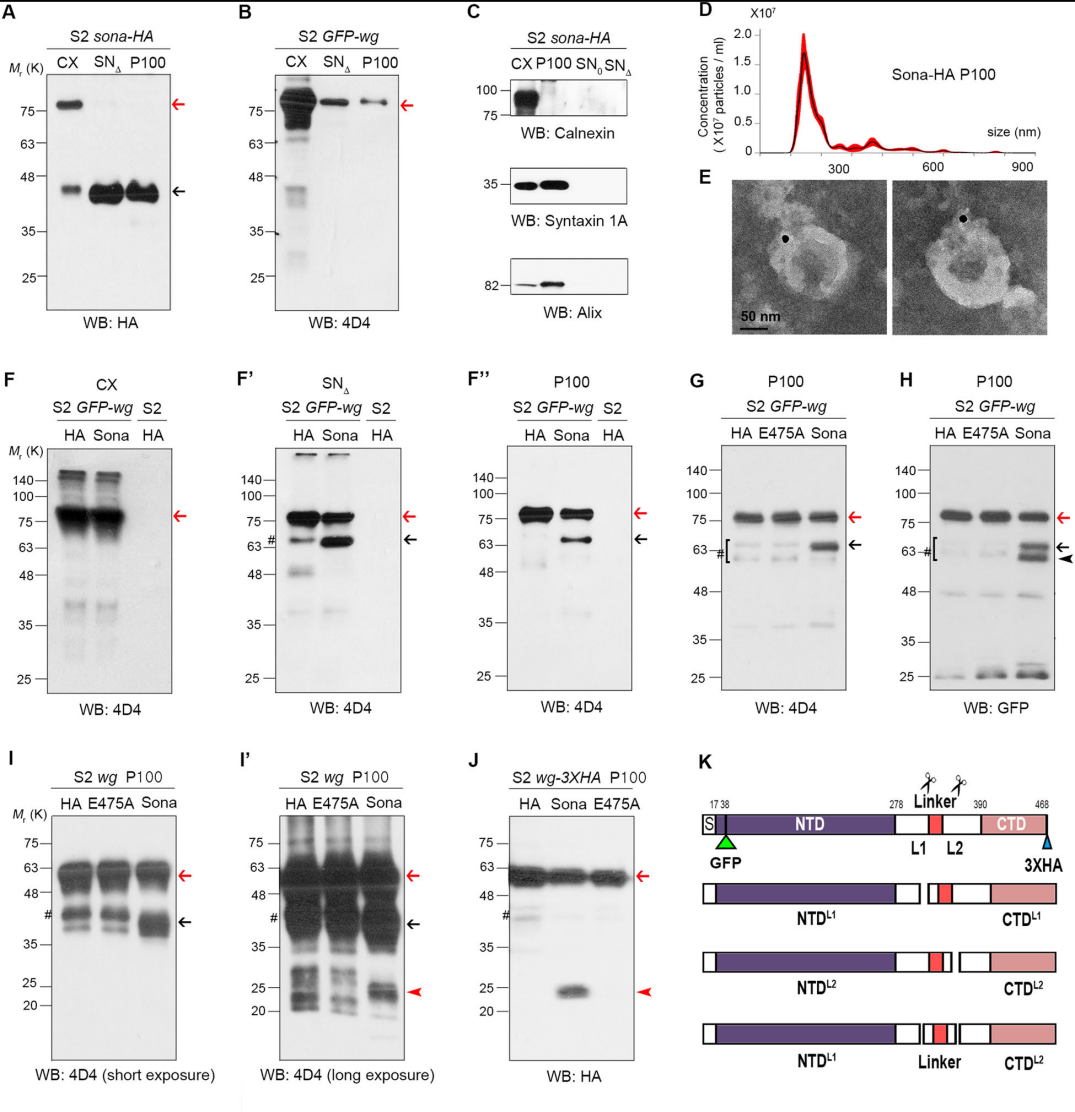
Active Sona is responsible for cleaving extracellular Wg in its linker region. In all western analyses, blotting antibodies are indicated at the bottom of each panel. Fractions and the source of cells transfected with different constructs are written at the top of panels. S2 cells are derived from a macrophage-like lineage of fly embryonic cells^78^, and express neither Sona nor Wg^10,19,33^. Therefore, *UAST-GFP-wg* and *UAST-sona-HA* cDNA constructs were expressed by actin-*Gal4* driver in S2 cells. HA is the preparation from cells transfected with the control *UAST-HA* vector. The pound signs (#) indicate the cleaved Wg fragments in the absence of Sona. These fragments may be generated by degradation during sample preparation or by some Wg-specific proteases endogenously expressed in S2 cells. **a** Full-length and processed forms of Sona-HA in cell extract (CX), SN_Δ_, and P100 fractions. The P100 fraction was four times more concentrated than the SN_Δ_ fraction, and the equal volumes of concentrated samples were loaded for the blot. Therefore, the actual amount of Sona in the SN_Δ_ fraction should be four times more than the one in the blot. The SN_Δ_ fraction always contained more Sona than the P100 fraction. Full-length Sona and active Sona are marked with red arrows and black arrow, respectively. **b** Full-length GFP-Wg (GFP-Wg^FL^) in CX, SN_Δ_, and P100 fractions. SN_Δ_ and P100 fractions were concentrated equally and the same volumes were loaded. GFP-Wg was more abundant in the SN_Δ_ fraction than the P100 fraction (red arrow)^19,33^. **c** Verification of CX, P100, SN_0_, and SN_Δ_ fractions using the ER marker Calnexin and the exosomal markers Alix and Syntaxin 1A (Syx1A). **d** Diameter and number of vesicles in P100 fractions from *S2 sona-HA* cells measured by NTA. **e** A immuno-EM image of Sona-HA in the P100 fraction with anti-HA antibody. The diameter of vesicles with Sona-HA is about 120 nm. **f**–**f”** GFP-Wg^FL^ (83 kDa) and a GFP-Wg fragment (65 kDa) are indicated with red and black arrows, respectively. **g**, **h** The black arrow and the arrowhead indicate 65 and 60 kDa GFP-Wg fragments, respectively. The same blot was used for both (**g**) and (**h**). **i** A blot in (**i**) was exposed longer in (**i’**). The 41 kDa and the 23 kDa fragments are marked by the black arrows and the red arrowhead, respectively. Non-tagged Wg^FL^ are marked by red arrows. **j** The 23 kDa Wg-3XHA fragment are marked with the red arrowhead. This fragment was readily detected because it has three times HA epitope than 4D4 epitope. **k** Domain structures of full-length and cleaved forms of Wg (modified from a report^79^). Two cleavage sites (L1, L2) are indicated with scissors. The 4D4 epitopes are marked with red

Sona is an ADAMTS protease secreted as an active form to the extracellular region^10^, and has biochemical and genetic interactions with Wg that is secreted by both conventional Golgi and exosomal transports^18–20^. As a first step toward understanding the role of extracellular Sona, we examined by which pathway Sona is secreted. To this end, we obtained conditioned media from the culture of *S2 sona-HA* cell line, and precleared it to obtain the initial supernatant fraction, SN_0_. Centrifugation of SN_0_ at 100,000×*g* yielded two fractions: a supernatant fraction (SN_Δ_) that contains soluble proteins secreted by Golgi transport, and a pellet fraction (P100) that contains exosomes^19,33^. The cell extract (CX) contained both full-length Sona-HA (red arrow) and the active form of Sona-HA lacking its pro-domain (black arrow), while the SN_Δ_ and P100 fractions contained only the active form of Sona-HA (Fig. 1a). Our data demonstrate that Sona is also secreted by both Golgi and exosomal transports (Fig. 1b). To prove further that Sona is secreted by exosomal pathway, we examined the P100 fraction of Sona-HA in detail. Purity of the P100 fraction was verified by the presence of the exosomal markers Syntaxin 1A (Syx1A) and Alix as well as the absence of the ER marker Calnexin^33,34^ (Fig. 1c). Particulate structures with 70–250 nm diameter were detected in the P100 fraction of S2 *sona-HA* or S2 *GFP-wg* by Nanoparticle tracking analysis (NTA), and Sona and Wg were present in the fraction with 1.09–1.11 g / mL density in sucrose step gradient^33^ (Fig. 1d and Supplemental Fig. S1A-B). Furthermore, Sona-HA was also present on the outer surface of vesicles (Fig. 1e and Supplemental Fig. S1C, D). To confirm the presence of Sona on exosomes in vivo, we examined whether Sona-HA and the exosomal marker CD63-GFP colocalize in wing discs. Unlike the lysosomal markers (Supplemental Fig. S2), half of CD63-GFP-positive (+) vesicles contained Sona-HA detected by both anti-HA and Sona-Pro antibodies (52.3 ± 10.3%, *n* = 65, Supplemental Fig. S1E)^10^, and about half of these CD63+ Sona+ vesicles contained endogenous Wg (21.5 ± 2.1%, *n* = 65, Supplemental Fig. S1F). These results demonstrate that Sona is present on exosomes.

### The active form of extracellular Sona is essential for cleavage of the Wg linker region

**Fig. 2:**
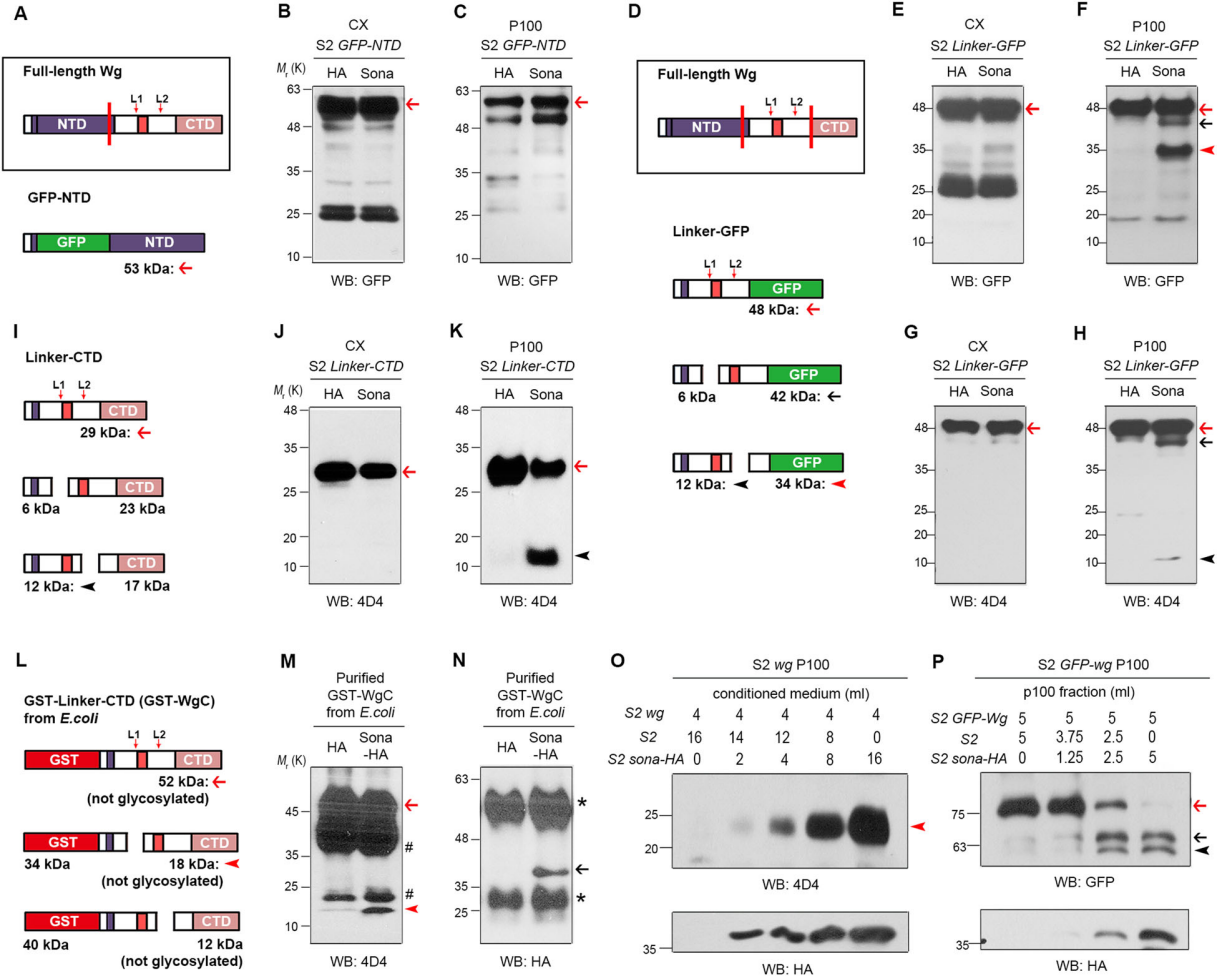
The linker region of Wg is necessary and sufficient for cleavage by Sona. A report on Wnt7a-NTD and Wnt7a-CTD constructs^42^ was used to select the site (red vertical line) for separating NTD and CTD. Domain diagrams of GFP-NTD in (**a**), Linker-GFP in (**d**), and Linker-CTD in (**i**), and putative cleaved products with calculated molecular weights and their corresponding protein bands in western blots are marked by arrows and arrowheads. No Sona-dependent cleavage occurred in CX (**b**, **e**, **g**, **j**). All uncleaved forms are marked with red arrows. **a**–**c** GFP-NTD generated from GFP-Wg^FL^ in (**a**) and western blots of GFP-NTD with or without Sona in (**b**–**c**). **d**–**h** Linker-GFP with and without Sona. Fragments of Linker-GFP cleaved at L1 and L2 sites are marked with black arrows and arrowheads in (**e**–**h**). **i**–**k** Linker-CTD with or without Sona. The black arrowheads indicate 12 kDa fragments generated by L2 cleavage. **l**–**n** In vitro assay for detection of cleaved Wg fragments from GST-linker-CTD expressed in *E. coil*. Black arrow indicates immunoprecipitated Sona-HA using anti-HA antibody and asterisks (*) indicate Rat IgG bands in (**n**). Red arrowhead indicates cleaved 18 kDa CTD^L1^ fragment in (**m**) and the pound signs (#) indicate the degraded product of GST-linker-CTD independent of active Sona. **o**, **p** The amount of cleaved Wg products increase proportionally to the amount of active Sona. Amounts of Wg and Sona are written on the top of panels. Precleared conditioned media prepared by centrifuging at 10,000×*g* were mixed and then the P100 fractions obtained from the mixture were analyzed in (**o**). 23 kDa fragments are marked by a red arrowhead in (**o**). P100 fractions from Wg-expressing S2 cells and those from Sona-expressing S2 cells were mixed, and then the P100 fractions obtained from those mixtures were analyzed in (**p**). 65 and 60 kDa GFP-Wg fragments are indicated by a black arrow and a black arrowhead, respectively in (**p**)

Coimmunoprecipitation of Sona and Wg^10^ suggested that Sona may cleave Wg. To test this, we examined whether any small Wg fragments are generated in the presence of Sona. In fact, a 65 kDa fragment smaller than 83 kDa full-length GFP-Wg (GFP-Wg^FL^) was detected by the 4D4 Wg antibody in both SN_Δ_ and P100 fractions only when Sona was coexpressed with GFP-Wg (black arrows in Fig. 1f’, f”). Such Sona-dependent changes were not detected in CX (Fig. 1f and Supplemental Fig. S3A, B). Interestingly, anti-GFP antibody detected both 65 kDa and an additional 60 kDa fragment (black arrowhead in Fig. 1h), indicating that these two fragments have the N-terminal region where GFP is inserted (Fig. 1k). Since these 65 and 60 kDa fragments were not produced by the protease-dead SonaE475A or in the presence of a zinc chelator, EDTA, metalloprotease activity of Sona is essential for Wg cleavage (Fig. 1g, h and Supplemental Fig. S3A-C). We located the two cleavage sites designated as L1 and L2 in the Wg linker based on two features. First, the 65 kDa but not the 60 kDa fragment has the 4D4 epitope (Fig. 1g, h). Second, the 4D4 epitope is located between amino acids 229 and 360 of Wg^35,36^. Cleavage at L1 and L2 should produce fragments named NTD^L1^, CTD^L1^, NTD^L2^, and CTD^L2^ (Fig. 1k). NTD^L1^ and NTD^L2^ are 60 kDa and 65 kDa fragments, respectively. Meanwhile, a 23 kDa fragment was detected with the 4D4 antibody when untagged Wg or GFP-Wg was coexpressed with Sona (red arrowheads in Fig. 1i’ and Supplemental Fig. S3A’), or detected with the HA antibody when Sona and Wg-3XHA with three HA tags at the C-terminus^37^ were co-expressed (red arrowhead in Fig. 1j). Therefore, this 23 kDa fragment is CTD^L1^ because it contains both the C-terminal part of Wg and the 4D4 epitope (Fig. 1k). However, CTD^L1^ fragments generated from the untagged Wg or GFP-Wg were detectable only after long exposure, and the intensity of CTD^L1^ fragment was much lower than that of NTD^L2^ (Fig. 1i-i’ and Supplemental Fig. S2A’). Moreover, the CTD^L2^ fragment was never detected when Wg-3XHA was coexpressed with Sona (Fig. 1j). The low levels of CTD^L1^ and the absence of the CTD^L2^ may be due to protein instability, which will be addressed in Fig. 2.

### The linker of Wg is necessary and sufficient for Wg cleavage by Sona

To examine whether the linker is the only region required for Wg cleavage, we generated two constructs, *GFP-NTD* and *linker-GFP* that encode 53 kDa GFP-NTD without the linker region and 48 kDa Linker-GFP with the linker region fused to GFP, respectively (Fig. 2a, d). When GFP-NTD and Sona-HA were coexpressed, Sona-dependent cleavage was not detected (Fig. 2c). In contrast, we found all cleavage products such as a 42 kDa L1 cleavage product (black arrows), a 34 kDa L2 cleavage product (red arrowheads), and a 12 kDa L2 cleavage product (black arrowheads) when Linker-GFP and Sona-HA were coexpressed (Fig. 2f, h). Furthermore, Wg cleavage by Sona occurred when Sona and Wg were prepared from different cells or different secretion pathways (Supplemental Fig. S3D-F). Therefore, the linker region is necessary and sufficient for Wg cleavage by Sona regardless of origin.

### The CTD domain of Wg-CTD fragments is responsible for protein instability

We previously mentioned that both CTD^L1^ and CTD^L2^ fragments might be unstable (Fig. 1i). However, the 42 kDa L1 Linker-GFP fragment that is equivalent to the 23 kDa Wg-CTD^L1^ fragment was readily detected (Compare Fig. 1i to Fig. 2h). This raised a possibility that the CTD domain itself is responsible for instability of Wg-CTD fragments. To test this, we generated *linker-CTD* that encodes 29 kDa Linker-CTD and compared its Sona-dependent cleavage products to those of Linker-GFP (Fig. 2i). When Linker-CTD and Sona were coexpressed, a 12 kDa 4D4-positive L2 cleavage product was detected (black arrowheads) but the expected 23 kDa CTD^L1^ was not detected even with multiple attempts (Fig. 2k). Moreover, the 34 kDa L2 linker-GFP fragment was detected (red arrowhead in Fig. 2f), which is equivalent to the 17 kDa Wg-CTD^L2^ fragment. As shown before, this 17 kDa Wg-CTD^L2^ fragment was never detected throughout this study (Fig. 1j, k). Therefore, both Wg-CTD fragments are less stable than their equivalents due to the CTD itself.

### Wg is directly cleaved by Sona

To test the enzyme-substrate relationship between Sona and Wg, we asked whether Sona cleaves Wg in vitro. Since the NTD of Wg is too hydrophobic to be expressed in *E. coli*, we generated GST-linker-CTD (Fig. 2l). We hypothesized that Sona may cleave GST-linker-CTD because the linker is sufficient for cleavage by Sona (Fig. 2l). When GST-linker-CTD purified from *E. coli* was incubated with active Sona purified from the SN_Δ_ fraction of S2 *sona-HA* (Supplemental Fig. S4A), the 4D4 antibody detected a Sona-dependent 18 kDa fragment (red arrowhead in Fig. 2m and Supplemental Fig. S4B; black arrow in Fig. 2n and Supplemental Fig. S4C). This 18 kDa fragment is the L1 cleavage product that is equivalent to the 23 kDa Wg-CTD^L1^ fragment, but is smaller because the CTD domain in the GST-linker-CTD is not glycosylated (Supplemental Fig. S4D). This suggests that Wg is a substrate of Sona.

If Sona directly cleaves Wg, the amount of cleaved Wg would positively correlate with that of Sona. To test this, the fixed amount of SN_0_ containing Wg was incubated with the increasing amounts of SN_0_ containing Sona-HA. As expected, the amount of Wg-CTD^L1^ fragment positively correlated with that of Sona (Fig. 2o). When the fixed amount of the P100 containg GFP-Wg was incubated with the increasing amounts of the P100 containing Sona-HA, the amount of Wg-NTD^L1^ and Wg-NTD^L2^ fragments also proportionally increased (Fig. 2p). This result is consistent with direct cleavage of Wg by Sona.

### Sona is required for cleavage of extracellular Wg in vivo

**Fig. 3:**
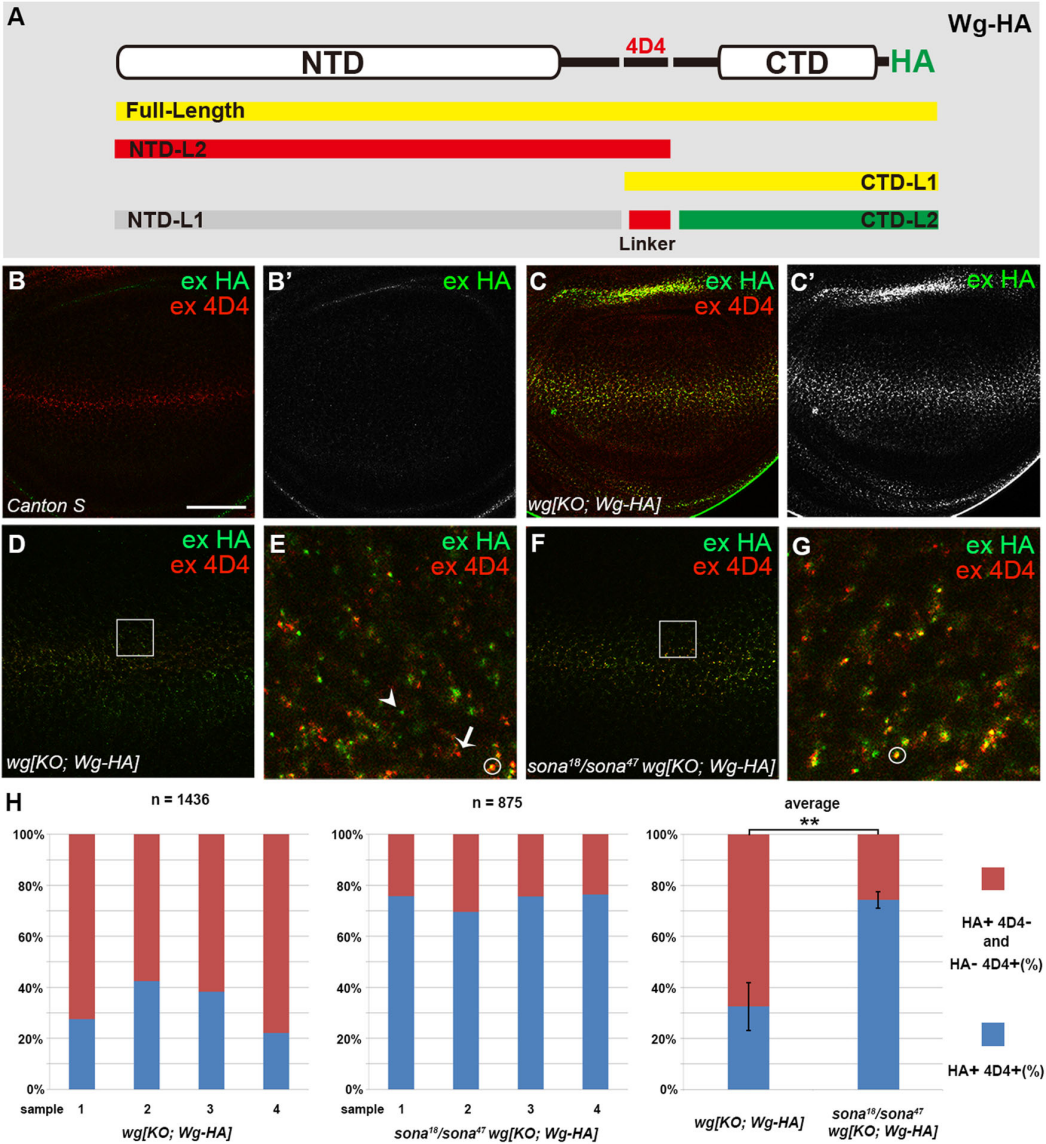
Cleaved Wg fragments are present in the basal ECM of wing discs. **a** Domain structures of Wg-HA and cleaved products drawn with red, green, and yellow bars. The Grey bar indicates undetectable NTD^L1^ fragment. The 4D4 epitope is present in the linker region. The HA (green) and 4D4 (red) signals match with the colors in the confocal images. **b**, **c** Extracellular staining of a homozygous *wg[KO;Wg-HA]* wing disc at the basal ECM. In the *wg[KO; Wg-HA]* strain, the endogenous *wg* gene is replaced by a *wg-HA* transgene that fully supports fly development^41^. Extracellular Wg-HA was detected by anti-4D4 and anti-HA antibodies. Extracellular HA signals were detected only in *wg[KO; Wg-HA]* (**c**) but not in *CS* wing discs (**b**). These two samples were stained at the same time under the same condition. **d**–**g** Extracellular 4D4 and HA staining of *wg[KO; Wg-HA]* and *sona*^*47*^*/sona*^*18*^ *wg[KO; Wg-HA]* wing discs. The *sona*^*18*^ *and sona*^*47*^ encode Sona with an internal deletion and a C-terminal truncation, respectively, and both cause pupal lethality^10^. Representative green (HA^+^ 4D4^–^ including CTD^L2^), red (HA^–^ 4D4^+^ including NTD^L2^ or the linker fragment), and yellow (HA^+^ 4D4^+^ including Wg^FL^ or CTD^L1^) signals are marked by an arrowhead, an arrow, and a circle, respectively. The squared regions in (**d**) and (**f**) are magnified in (**e**) and (**g**), respectively. **h** The proportions of blue bars representing HA^+^4D4^+^ structures and red bars representing both HA^+^4D4^–^ and HA^–^ 4D4^+^ structures. 4 *wg[KO;Wg-HA]* discs and 4 *sona*^*47*^*/sona*^*18*^ *wg[KO; Wg-HA]* discs were used for counting extracellular structures. The total numbers of counted structures are written on the top of the graph. The average percentages of counted vesicles are shown on the right. Graphs are displayed as mean ± S.E.M, where ^∗∗^*p* < 0.01. Scale bars for (**b**) and (**c**), 60 μm; (**d**) and (**f**), 40 μm; (**e**) and (**g**), 5 μm

We next asked whether cleavage of extracelluar Wg also occurs in vivo. Wg is highly expressed along the DV midline of wing discs^38–40^. To detect cleaved Wg forms, we examined extracellular Wg-HA pattern in the DV midline of *wg[KO; Wg-HA]*^41^. Assuming Sona cleaves Wg at L1 and L2 cleavage sites in vivo, we expected to detect extracellular structures including Wg-HA^FL^ and four additional Wg fragments except NTD^L1^ in *wg[KO; Wg-HA]* wing discs using anti-HA and 4D4 antibodies (Fig. 3a). We found not only yellow structures (HA^+^ 4D4^+^) that contain full-length Wg but also the green (HA^+^ 4D4^–^) and the red structures (HA^–^ 4D4^+^) that represent cleaved Wg fragments (Fig. 3a, e). These cleaved structures were also detected in *wg* > *GFP-wg* discs (Supplemental Fig. S5). Thus, cleaved Wg fragments were present in the extracellular region.

If Sona plays a major role in the cleavage of Wg^FL^, *sona* mutants should have more Wg^FL^ than wild-type. To test this, we compared the percentage of yellow structures (HA^+^ 4D4^+^) in *sona*^*18*^*/ sona*^*47*^ *wg[KO; Wg-HA]* discs with that in control *wg[KO; Wg-HA]* discs (Fig. 3d–h). While 74.1% (648/875) were yellow in *sona*^*18*^*/sona*^*47*^ discs, only 32.0% (460/1436) were yellow in wild-type discs (Fig. 3h). This suggests that Wg cleavage occurred at a lesser extent in the *sona* discs. This supports that Sona is a major player in cleavage of Wg in vivo.

### Prolonged overexpression of Wg-CTD induces morphological defects and lethality

We reasoned that at least one of the cleaved forms of Wg should be active because Sona positively regulates Wg signaling^10^. To test which Wg fragment is active, we performed luciferase assay in S2R+ cells that expressed Wg-NTD or Wg-CTD. Neither Wg-NTD nor Wg-CTD, however, showed any Wg activity (Supplemental Fig. S6A). This was unexpected because artificially engineered Wnt7a-CTD is reported to be active in TopFlash reporter assay^42^. It is possible that S2 R+ cells lack some essential components that are required for Wg-CTD activity^43^, or Wg-CTD is too unstable in S2 R+ cell culture (Supplemental Fig. S6). Instead of finding the better condition for luciferase assay, we decided to test the activity of Wg fragments in vivo with *UAS-GFP-wg-NTD* and *UAS-wg-mycCTD* transgenic flies (Fig. 2a and Supplemental Fig. S7A).

**Fig. 4:**
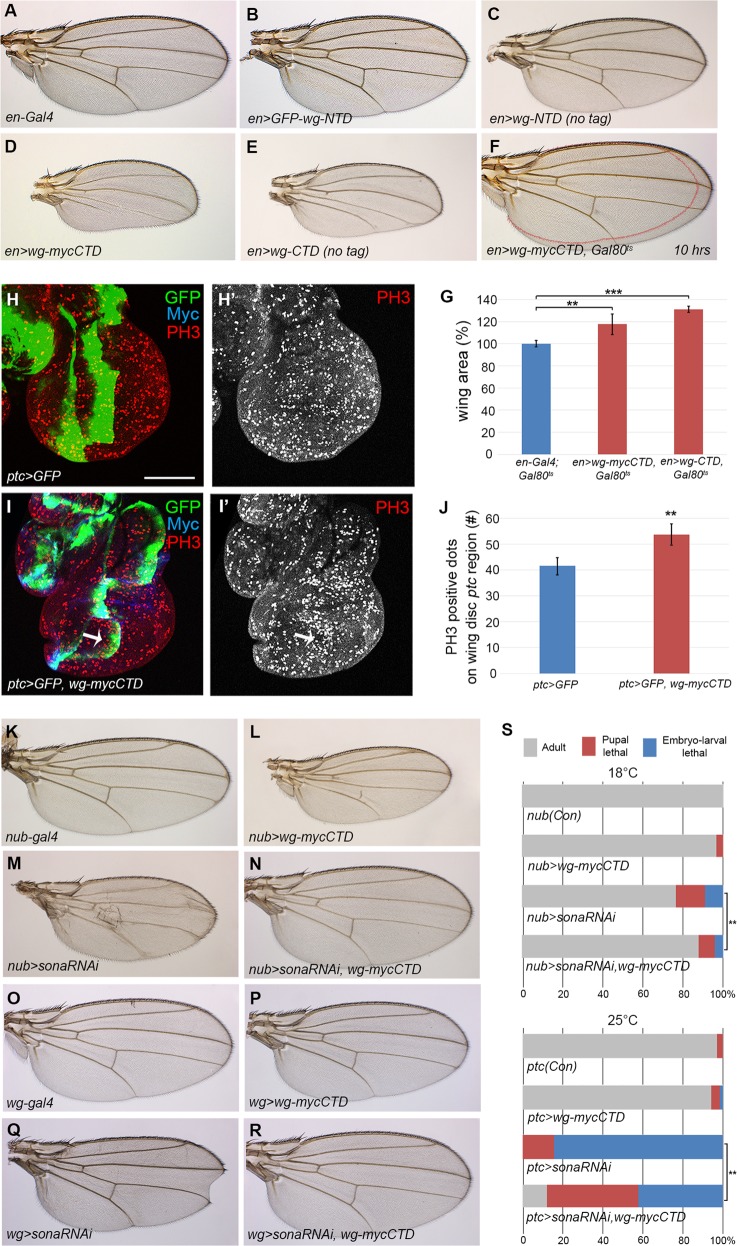
Wg-CTD but not Wg-NTD is functional in vivo. The genotypes of female wings are indicated at the lower left of each panel. **a** The *en-Gal4* wing at 18 °C as a control. **b**, **c** Both *en* *>* *GFP-wg-NTD* wing in (**b**) and *en* *>* *wg-NTD* (no tag) wing in (**c**) were normal. **d**, **e** prolonged expressions of Wg-CTD in *en* *>* *wg-mycCTD* flies (**d**) and *en* *>* *wg-CTD* (no tag) flies (**e**) induce deformed wings with smaller posterior region than the control wing in (**a**) similar to phenotypes by prolonged expression of Wg^48,49^. **f** *en* *>* *wg-mycCTD, Gal80*^*ts*^ wings from flies cultured for 10 h at 30 °C during the mid-third instar stage for transient Wg-mycCTD expression. Outline of the control wing in (**a**) is drawn in (**f**). **g** The average wing size of *en* *>* *wg-mycCTD, Gal80*^*ts*^ and *en* *>* *wg-CTD, Gal80*^*ts*^ flies (*n* = 31 each) cultured for 10 h at 30 °C are shown in bar graphs as mean ± S.E.M. **h**, **i** PH3 pattern in mid-3rd instar wing discs of *ptc* *>* *GFP* and *ptc* *>* *GFP, wg-mycCTD*. An arrow indicates increase in PH3 staining along the *ptc* region in (**i**). **j** The graph represents the average number of PH3 positive signals in the *ptc* region of *ptc* *>* *GFP* and *ptc* *>* *GFP, wg-mycCTD* wing discs (*n* = 10). **k**–**r** Wg-mycCTD expression rescues *sona RNAi* phenotypes. All *nub* *>* *sona RNAi* wings are small and malformed in (**m**). Wing phenotypes of *nub* *>* *sona RNAi* were rescued by coexpression of Wg*-*mycCTD in (**n**). *wg* *>* *wg-myc-CTD* wings in (**p**) did not show any phenotype similar to the control *wg-Gal4* wings in (**o**). *wg* *>* *sona RNAi* (*n* = 71) had notched wing phenotype in (**q**), but *wg* *>* *sona RNAi, wg-mycCTD* had normal wings (*n* = 68) in (**r**). **s** Wg-mycCTD expression partially suppressed the lethality induced by *sona RNAi*. Adult flies, pupal lethal and embryonic to larval lethal in percentages are drawn in colored bars. 23% of *nub* *>* *sona RNAi* flies were embryonic to pupal lethal, but only 12% of *nub* *>* *sona RNAi, wg-mycCTD* flies were embryonic to pupal lethal (*n* = 160 each). Similarly, none of *ptc* *>* *sona RNAi* flies but 12% of *ptc* *>* *sona RNAi*, *wg-mycCTD* flies (*n* = 140) survived to adulthood and their lethal stage was also significantly delayed from embryonic to pupal stage (Supplemental Fig. S7A-D). Scale bar, 100 μm, ∗∗*p* < 0.01; ∗∗∗*p* < 0.001

Overexpression of GFP-Wg-NTD with *engrailed* (*en*)*-Gal4* or *nubbin* (*nub*)*-Gal4* produced no phenotypes (Fig. 4b and Supplemental Fig. S7B). In contrast, *en* > *wg-mycCTD* and *nub* > *wg-mycCTD* wings were small and deformed (Fig. 4a, d, k, l), and the posterior region of *en* *>* *wg-mycCTD* wing discs were smaller than that of control discs (Supplemental Fig. S7K, L). Furthermore, *cubitus interruptus (ci)* *>* *wg-mycCTD* eyes were small and rough (Supplemental Fig. S7N, O). Expression of Wg-mycCTD by *actin-Gal4* or *tubulin-Gal4* induced early larval lethality (*n* > 40) but that of GFP-*wg*-NTD produced no lethality. Expression of the untagged Wg-NTD and Wg-CTD also generated phenotypes similar to the tagged counterparts (Fig. 4c, e and Supplemental Fig. S7A, E, F, J, M). Taken together, Wg-CTD but not NTD is an active Wg form in vivo.

### Transient overexpression of Wg-CTD stimulates cell proliferation

Wg stimulates cell proliferation as a mitogen^44,45^, and moderate Wg overexpression increases the number of phosphohistone 3 (PH3)-positive cells^44,46^. To check whether Wg-CTD also induces cell proliferation, we transiently expressed Wg-CTD using *Gal80*^*ts*^ in order to avoid cell death or cell cycle arrest by prolonged Wg signaling^47–49^. Indeed, expression of Wg-mycCTD or untagged Wg-CTD for ten hours increased wing size by 17.6 and 31.3%, respectively (*n* = 31, Fig. 4f, g). Likewise, the number of PH3-positive cells was increased along the *ptc* region compared to control *ptc* *>* *GFP* discs (Fig. 4h–j). Therefore, Wg-CTD is able to promote cell proliferation.

### Wg-CTD rescues the loss of *sona* phenotypes

Wg-CTD would rescue the loss-of-function phenotypes of extracellular Sona if generation of Wg-CTD is a main function of extracellular Sona. To test this, *sona RNAi-1*^*11-4*^ (*sona RNAi*, hereafter) was coexpressed with Wg-mycCTD and the wing phenotype and lethality were compared to those of sole *sona RNAi* expression^10^. All *nub* *>* *sona RNAi* wings were small, wrinkled or both^10^ (*n* = 95, Fig. 4k, m) but 68% of *nub* *>* *sona RNAi*, *wg-mycCTD* wings were normal (*n* = 109, Fig. 4n, s). Notched wing phenotype was observed in 55% of *wg* *>* *sona RNAi* (*n* = 71) but only in 6% of *wg* *>* *wg-mycCTD,* *sona RNAi* (*n* = 68) flies (Fig. 4o–r). Furthermore, notched wing phenotype of *ptc* *>* *wg-mycCTD* was rescued in *ptc* *>* *sona RNAi*, *wg-mycCTD* flies (Supplemental Fig. S8E-G). Therefore, Wg-CTD expression rescued loss of *sona* phenotypes.

### Wg-CTD activates canonical Wg signaling

**Fig. 5:**
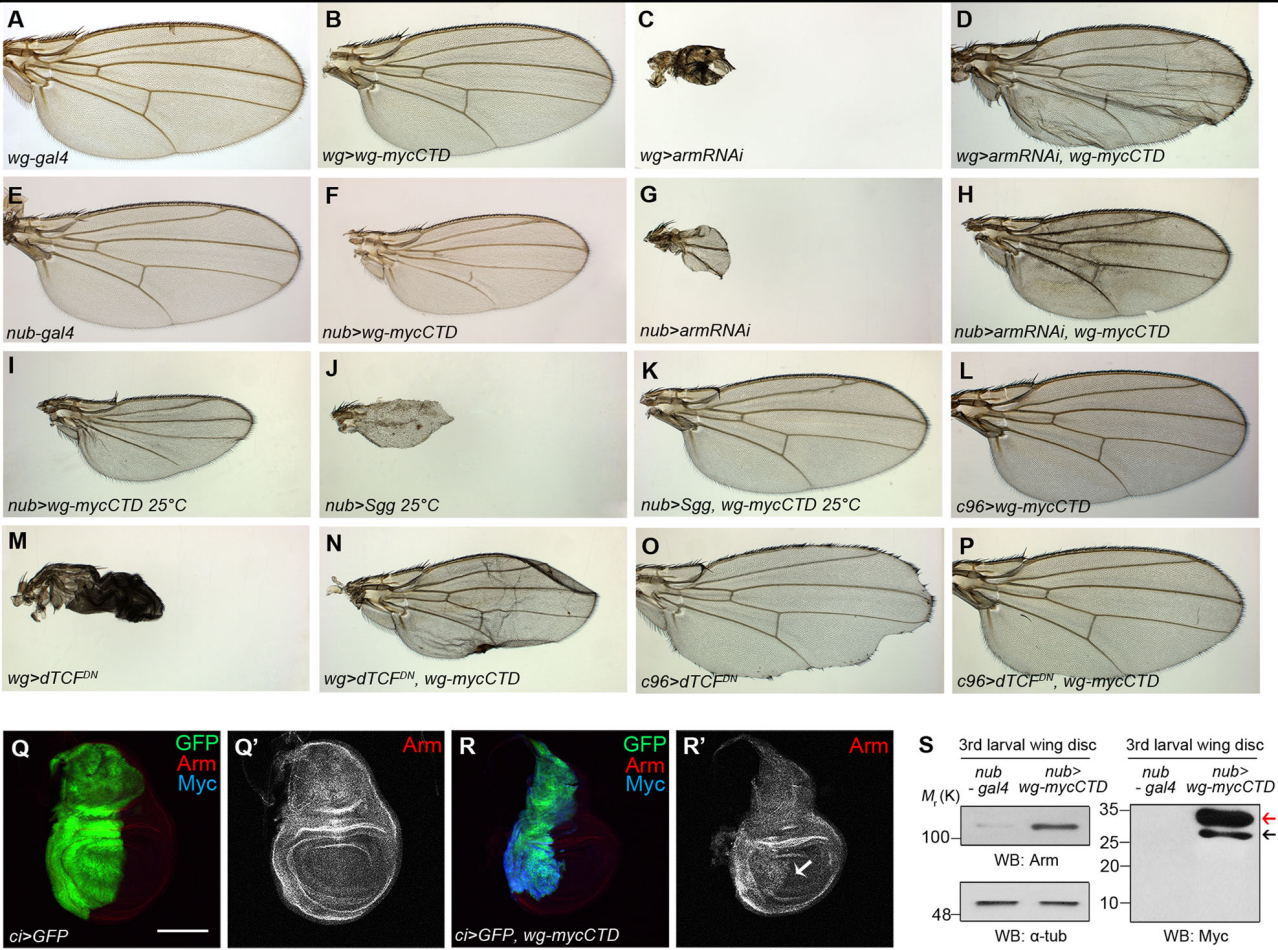
Wg-CTD activates canonical Wg signaling. **a**–**p** Adult female wings are shown with their genotypes indicated at the lower left of each panel. All flies were cultured at 18 °C except flies in (**i**), (**j**) and (**k**) cultured at 25 °C. The *wg-Gal4* in (**a**) and *nub-Gal4* in (**e**) wings as controls. The *wg* *>* *wg-mycCTD* wings were normal in (**b**), and the *nub* *>* *wg-mycCTD* wings were smaller in (**f**). The *wg* *>* *arm RNAi* flies in (**c**) and *nub* *>* *arm RNAi* flies in (**g**) were mostly pupal lethal but a few escapers (<1%) had severely malformed and small wings (*n* > 150). Co-expression of Wg-mycCTD rescued the wing size and morphology in (**d**) *wg* *>* *arm RNAi, wg-mycCTD* flies (2 out of 48) and (**h**) *nub* *>* *arm RNAi, wg-mycCTD* flies (8 out of 58). The *nub* *>* *Sgg* wings in (**j**) were small, and were rescued to normal wings by Wg-CTD co-expression (19 out of 81) in (**k**). The *wg* *>* *dTCF*^*DN*^ flies in (**m**) (*n* > 100) were 100% pupal lethal with severely malformed wings but the *wg* *>* *dTCF*^*DN*^*, wg-mycCTD* wings were larger (3 out of 26) in (**n**). The *c96* *>* *dTCF*^*DN*^ flies in (**o**) had notched wings but *c96* *>* *dTCF*^*DN*^, *wg-mycCTD* flies were normal (37 out of 50) in (**p**). **q**, **r** Arm in the wing discs of *ci* *>* *GFP* in (**q**) and *ci* *>* *GFP, wg-mycCTD* in (**r**) larvae. An arrow in (**r’**) indicates the anterior region where Arm is increased. **s** Western analysis to detect Arm in the CX prepared from *nub* *>* *wg-mycCTD* wing discs. α-Tubulin was used as a loading control. A red arrow indicates full-length Wg-mycCTD, and a black arrow indicates a cleaved fragment that may be equivalent to the L1 cleavage product of Wg-mycCTD. As previously shown, Wg-CTD L2 fragment was not detected. Scale bar, 100 μm

Cytoplasmic Arm becomes stabilized by activation of canonical Wg signaling^50^. Because Wg-CTD expression rescued the lethality and wing defects induced by *arm RNAi*, *Sgg* or *dTCF*^*DN*^ expression (Fig. 5a–p), we tested whether Wg-CTD stabilizes the cytoplasmic Arm. In fact, *ci* *>* *GFP, wg-mycCTD* wing discs had the increased level of Arm in the anterior region (Fig. 5q, r). More Arm was also present in the CX of *nub* *>* *wg-mycCTD* compared to control *nub-Gal4* wing discs (Fig. 5s).

**Fig. 6:**
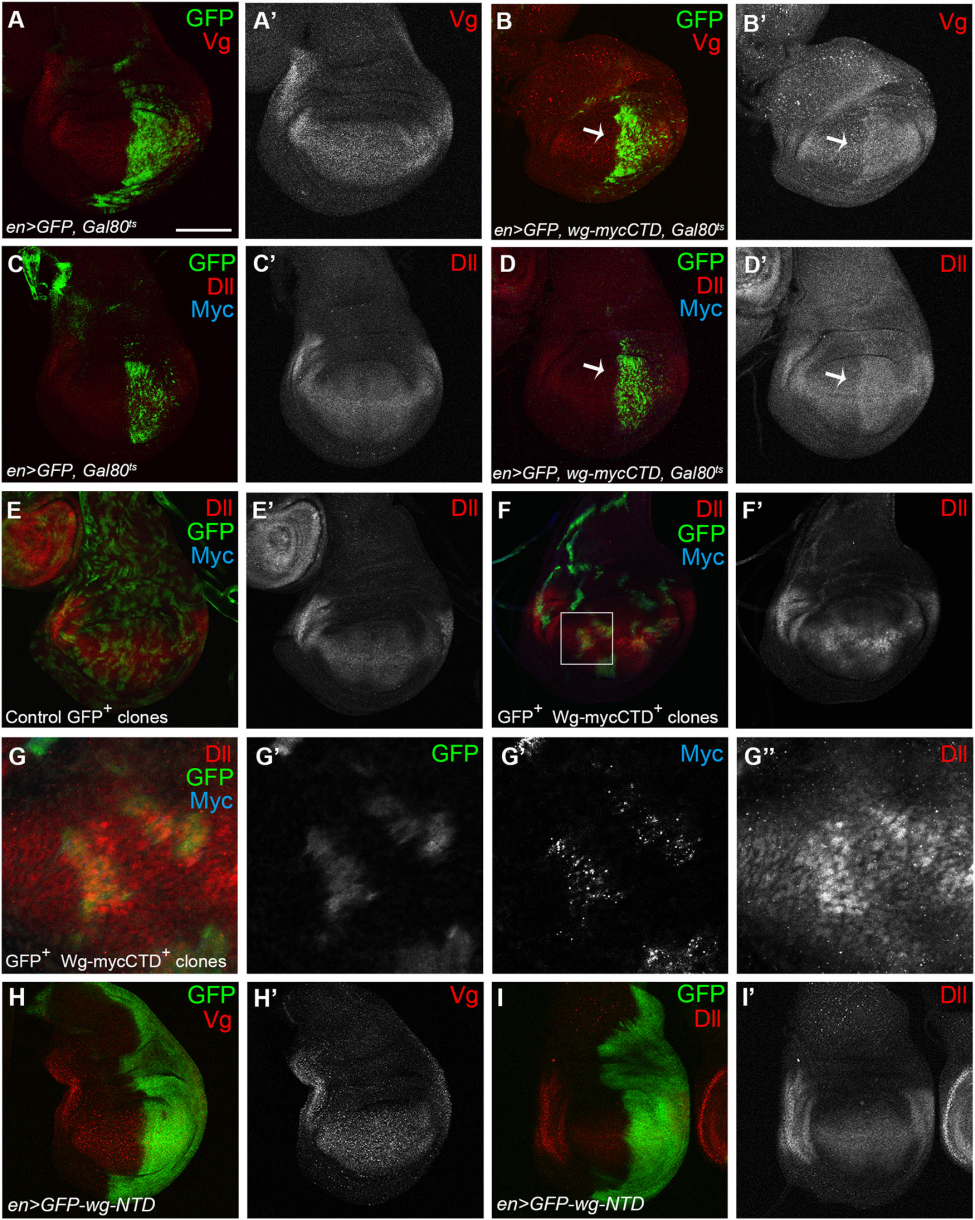
Wg-CTD increases the level of Dll and Vg. **a**–**d** The flies were shifted from 18 °C to 30 °C for 10 h during the late second and early third larval instar for transient expression. The *en* *>* *GFP, Gal80*^*ts*^ discs did not show any change in Vg and Dll in (**a**) and (**c**), but the *en* *>* *GFP, Gal80*^*ts*^*, wg-mycCTD* discs showed increased Vg and Dll in the posterior region marked by arrows in (**b**) and (**d**). **e**–**g** Analysis of GFP^+^ clones that overexpress Wg-CTD in *hsflp; actin* *>* *y* *>* *Gal4; UAS-GFP* (**e**) and *hsflp; actin* *>* *y* *>* *Gal4; UAS-GFP/UAS-wgmycCTD* (**f**) flies. GFP^+^ control clones in (**e**) showed no change but GFP^+^ Wg-CTD^+^ clones in (**f**) had higher level of Dll. The boxed region in (**f**) is magnified in (**g**). **h**, **i** *en* *>* *GFP-wg-NTD* wing discs showed no change in the levels of Vg in (**h**) or Dll in (**i**). Scale bar, 100 μm except (**g**), 40 μm

We next examined whether Wg-CTD increases levels of Wg effector proteins, Vg and Dll that are induced by canonical Wg signaling^51–54^. Transient expression of Wg-CTD by *en-Gal4* increased the level of Vg and Dll in the posterior region of wing discs (Fig. 6a–d), and Wg-CTD-expressing flp-out clones had higher level of Dll (Fig. 6e–g). The untagged Wg-CTD expression in *en* *>* *wg-CTD* discs also increased the level of Dll (Supplemental Fig. S9B). GFP-Wg-NTD and untagged Wg-NTD failed to change the level of Wg effector proteins (Fig. 6h, i and Supplemental Fig. S9A). Therefore, Wg-CTD is a new form of active Wg that induces canonical Wg signaling.

### Wg-CTD upregulates the level of Cyc D for cell proliferation

**Fig. 7:**
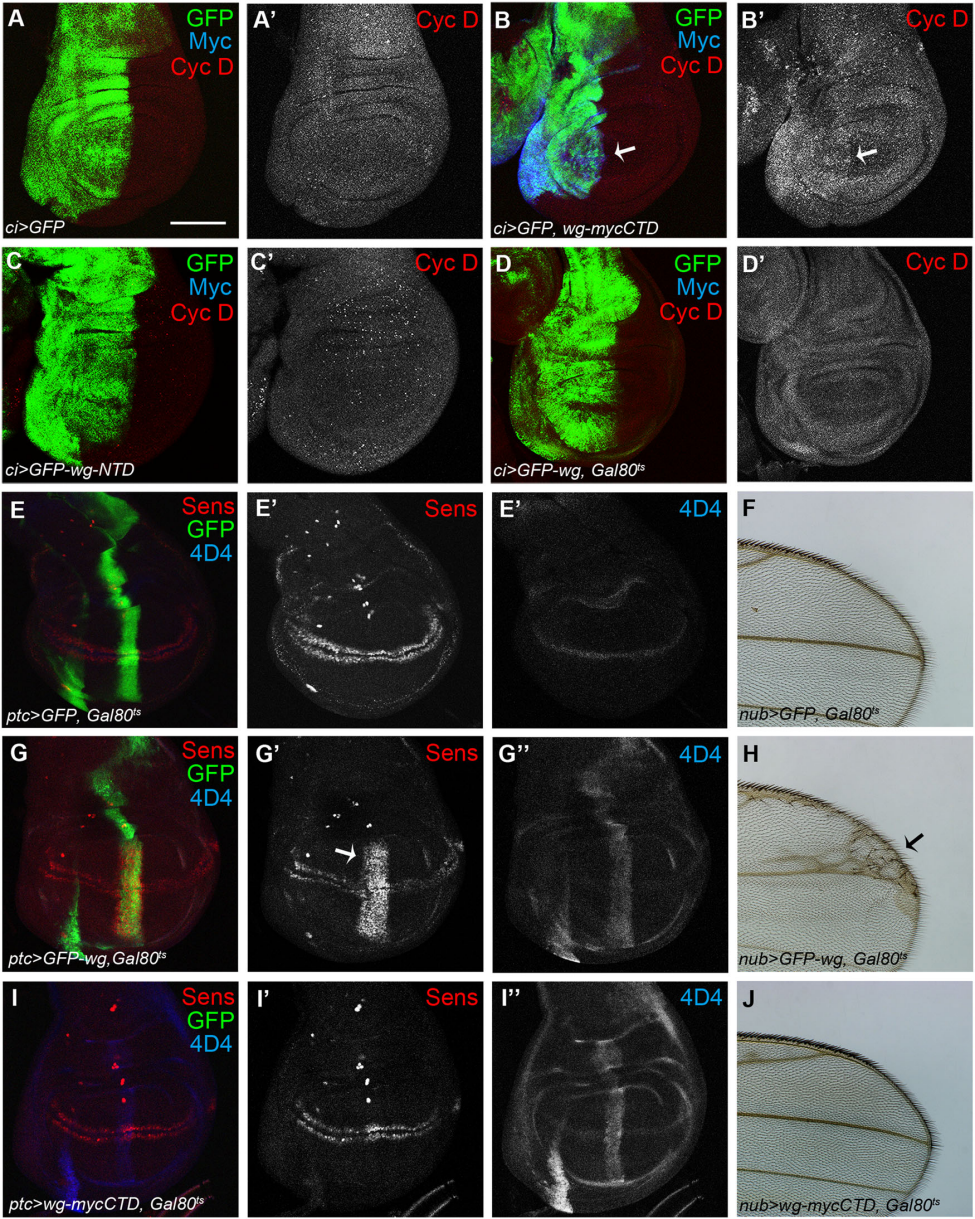
Wg-CTD may be specialized for cell proliferation than differentiation. Transgenes were transiently expressed by shifting the culture temperature to 30 °C for 12 h during the late second and early third larval instar in all cases. **a**–**d** The level of Cyc D in wing discs. Arrows mark the region with higher level of Cyc D in *en* *>* *GFP, wg-mycCTD* discs in (**b**). No changes in control *ci* *>* *GFP* in (**a**), *en* *>* *GFP-wg-NTD* in (**c**) and *en* *>* *GFP-Wg, Gal80*^*ts*^ in (**d)** discs**. e**–**j** Sens expression in wing discs and sensory bristle formation in adult wings. Sens level in control (**e**) and *ptc* *>* *wg-mycCTD, Gal80*^*ts*^ wing discs in (**i**). The arrow indicates ectopic Sens by GFP-Wg^FL^ in *ptc* *>* *GFP-wg, Gal80*^*ts*^ wing discs in (**g**). Expression of full-length GFP-Wg in (**h**) induced ectopic bristles in wing blades, but control in (**f**) and Wg-CTD expression in (**j**) did not induce any ectopic bristles. Scale bar, 100 μm

Overexpressed Wg^FL^ partially rescued the lethal phenotype of *wg* mutants but Wg-CTD did not (Supplemental Fig. 9C-E). This demonstrates that Wg-CTD can carry out only subsets of Wg signaling and so called ‘Wg signaling’ is induced by combined activity of both Wg^FL^ and cleaved Wg-CTD. An important question is then whether Wg-CTD has any unique functions unshared by Wg^FL^. We hypothesized that Wg-CTD may be more specialized for cell proliferation than Wg^FL^ because Sona increases the level of Cyc D^13^. Indeed, Cyc D was upregulated by prolonged expression of Wg-CTD in the anterior region of *ci* *>* *wg-mycCTD* wing discs and by transient expression of Wg-CTD in the posterior region of *en* *>* *wg-mycCTD, Gal80*^*ts*^ discs cultured for 12 h at 30 °C (Fig. 7a, b; Supplemental Fig. S9F, G). GFP-Wg^FL^ or GFP-Wg-NTD expression, however, did not change the level of Cyc D (Fig. 7c, d). Taken together, Wg-CTD is able to induce Cyc D. Wg signaling plays an important role in neuronal differentiation by inducing *sens* in the DV margin of wing discs^55–57^. Transient expression of GFP-Wg increased the level of Sens in *ptc* *>* *GFP-wg, Gal80*^*ts*^ discs, and induced ectopic sensory bristles in *nub* *>* *GFP-wg, Gal80*^*ts*^ wings, which are consistent with previous reports^58,59^ (Fig. 7e–h). In contrast, transient expression of Wg-CTD did not induce ectopic Sens in *ptc* *>* *wg-mycCTD Gal80*^*ts*^ and *en* *>* *wg-mycCTD Gal80*^*ts*^ wing discs (Fig. 7i and Supplemental Fig. S9I). Wings of *nub* *>* *wg-mycCTD, Gal80*^*ts*^, *nub* *>* *wg-mycCTD*, and *nub* *>* *wg-NTD* flies also had no ectopic bristles (Fig. 7j and Supplemental Fig. S9K-L). Thus, Wg-CTD is not able to induce Sens unlike Wg^FL^.

## Discussion

We report here that Sona cleaves extracellular Wg into Wg-NTD and Wg-CTD, and the Wg-CTD is a new form of active Wg (Supplemental Fig. S10). Because Wg-CTD substantially rescued the *sona* loss-of-function phenotypes such as lethality and wing defects (Fig. 4), generation of Wg-CTD seems to be one of Sona’s major functions. Wnt modifications such as lipidation and glycosylation have been extensively studied, but Wnt cleavage has not been addressed except for the *Xenopus* Tiki protease. Tiki reduces Wnt secretion by cleaving the amino-terminal region of intracellular Wnt that is required for the lipidation of Wnt^60^. While Tiki aims to decrease the amount of secreted Wnt, Sona aims to generate a new active form of Wg from an already active Wg^FL^.

Genetic interaction between *wg-CTD* and other Wg signaling components indicates that Wg-CTD activates Wg signaling similar to Wg^FL^ (Fig. 5). However, there are several differences between these two forms of Wg. First, Wg-CTD but not Wg^FL^ increased the level of Cyc D (Fig. 7). Overexpressed Cyc D-Cdk4 in flies accelerates cell division of undifferentiated cells such as wing disc cells^61^. Sona also induces Cyc D and promotes cell proliferation in a cell non-autonomous manner^13^. Therefore, Wg-CTD generated by extracellular Sona seems to induce Cyc D in the neighboring cells for cell proliferation. Second, both Wg-CTD^L1^ and Wg-CTD^L2^ are less stable than Wg^FL^. Instability of Wg-CTD may be an essential feature because mitogens and their downstream components are often removed by degradation to prevent excessive cell proliferation^62^ (Fig. 4). Presence of Wg-CTD^L2^-like structures in wing discs (Fig. 3), however, implies that these Wg-CTD^L2^-like structures may be stabilized *in vivo* by ECM components to achieve spatiotemporal regulation of the mitogenic activity^63,64^. Third, Wg-CTD is not able to induce Sens (Fig. 7 and Supplemental Fig. S8). Sens expression in the DV midline is required for differentiation of wing margin bristles^55–57^, unlike Vg that is essential for cell proliferation and cell survival^23,65^.

The difference between the two Wg forms in Sens induction may be due to their differential affinity to Fz receptors, based on the report that NTD and CTD of vertebrate Wnts able to interact Fz receptors independently from each other with different affinity^66^. It has been proposed that Wnt is generated during evolution via the fortuitous fusion of two ancestral proteins analogous to its NTD, homologous to a class of lipid-interacting proteins, and CTD, homologous to a group of cytokines involved in cell signaling^42,66,67^. This explains why NTD mutants are unable to be secreted^68^, while CTD mutants are secreted but inactive^69^. Given the evolutionary conservation of the components of Wnt signaling, ADAMTSs may also be involved in the generation of functional Wnt-CTD in mammals^70,71^. We expect that further study on the relationship between Wnts and ADAMTSs will expand our understanding on Wnt signaling and Wnt-related diseases.

## Materials and methods

### *Drosophila* strains, transgenic lines and generation of ectopic clones

*sona* mutants, *sona RNAi* lines, *UAS-sona*, and *UAS-sona-HA* are described elsewhere^10^. The *UAS-wg-mycCTD, UAS-GFP-wg-NTD, UAS-wg-CTD* and *UAS-wg-NTD* flies were generated for this study. *UAS-CD63-GFP*^72^, *UAS-GFP-wg*^73^, *wg[KO; Wg-HA]*^41^, *UAS-GFP-lamp*^74^, *wg*^*Gal4*^^75^ and *ci-Gal4*^76^ were kindly provided by other labs that produced them. All other lines were obtained from the Bloomington stock center.

### DNA constructs

The *pAc-GFP-wg* and *pAc-wg-3XHA* were constructed by recombining the *pAc5.1* vector with *GFP-wg* or *wg-3XHA* obtained from *MK33-GFP-wg* (a gift from J.P. Vincent, unpublished) or *UAS-wg-3XHA*^37^. To generate the *GFP-wg-NTD (GFP-NTD)* and *wg-mycCTD* constructs, a *myc* tag was inserted in the DNA corresponding to the region between Arg367 and Tyr368 in GFP-Wg. DNA fragments representing GFP-NTD (1–245) and mycCTD (1–22, 245–468) were then amplified by PCR and inserted into *pUAST* vectors by recombination cloning methods.

### Cell lines, cell culture, and exosome preparation

*Drosophila* S2 *tub-wg*, S2R+, and S2 cell lines were obtained from DGRC. S2 *GFP-wg, wg-3XHA*, and *sona-HA* stable cell lines were generated by selection under 2.5 μg/ml hygromycin B (Invitrogen) as follows. *Drosophila* S2 cell were grown in M3 media (Sigma-Aldrich) supplemented with 10% IMS (Sigma-Aldrich) at 25 °C. Stable cell lines were grown with hygromycin in 10% IMS M3 media, and S2 *tub-wg* cells were cultured in 10% FBS M3 media. Transfections were carried out with Effectene (Qiagen) or Cellfectin (Invitrogen) according to the manufacturers’ instructions. For exosome preparation, 7–40 ml of conditioned media obtained from cultures (1.25 × 10^6^ cells / ml) were used as described^19^. The size and number of the exosomes in the P100 fraction were measured by Nanosight NC300 (Malvern Instruments).

### Immunocytochemistry and Western analysis

Fly larvae were cultured at 25 °C unless stated otherwise. Wing discs from the late third instar larvae were used for intracellular staining and extracellular staining^24^. For immunocytochemistry, we used Sona-Pro, 1:300–500; Golgi (Calbiochem, mouse), 1:200; GFP (Abd serotec, sheep), 1:100; Senseless (a gift from H. Bellen, guinea pig), 1:1000; HRS (a gift from H. Bellen, guinea pig), 1:1000; WgN (sc-28646 Santa Cruz, rabbit), 1:100; Wg (DSHB, mouse), 1:1000; HA (Roche, rat), 1:300; HA (Santa Cruz, rabbit), 1:300; Vg (gift from Sean B. Carroll, rabbit), 1:100; Wg (DSHB, mouse), 1:100; Dll (Santa Cruz, goat), 1:100. For the extracellular staining of proteins, we used 10 times more antibodies than for the intracellular staining. Fluorescent images were captured using a Zeiss LSM laser scanning confocal microscope and processed with Adobe Photoshop.

Western analysis was carried out as described^10^. For western analysis, we used Sona-Pro (our lab, rabbit), 1:5000; HA (Santa Cruz, rabbit), 1: 250; GFP (Abcam, rabbit), 1:10,000; Wg (DSHB, mouse), 1:500–1000; Syntaxin 1A (DSHB, mouse), 1:25; Alix (gift from T. Aigaki, mouse), 1:500; Actin (DSHB, mouse), 1:500; Calnexin (gift from N.J. Colley, rabbit), 1:2000.

### Electron microscopy

For immunogold labeling, P100 fraction from S2 *sona-HA* cells were plated on grids, blocked with 5% BSA in PBS and incubated with anti-HA antibody (1:5). Then, samples were washed with 0.1% BSA in PBS and incubated in secondary anti-rabbit antibody conjugated with 15 nm gold particles (AURION). After 8 times wash with PBS for 5 min each, samples were incubated in 1% glutaraldehyde for 5 min. Then, samples were washed with H_2_O for 8 times before staining with Phosphotungstic acid (PTA). Sample grids were air-dried completely and visualized using a transmission electron microscope (Talos F200X).

### In vitro GST-Wg cleavage assay

For purification of GST-Wg, pGEX-4T-1-WgCterm was expressed in BL21 *E. coli* strain. Then, we purified the GST-Wg protein by standard column-based protocols (GST-column, 1^st^ SP Sepharose column, 2^nd^ SP Sepharose column). For purification of active Sona from S2 cell culture, CX and SN_Δ_ fractions were prepared and lysis buffer without EDTA and Protein inhibitor cocktail (PIC) were added to these fractions. Active Sona was obtained by mixing with HA-conjugated bead and precipitating the beads. GST-Wg and active Sona were mixed and incubated at 25 °C overnight.

### Sucrose step gradient

Exosome pellets were resuspended in 0.25 M sucrose and loaded on top of a sucrose step gradient before being centrifuged at 100,000xg in a Beckman SW41Ti rotor for 3 h as described^77^. Ten to twelve fractions of 1 mL each were then manually collected from the bottom of the gradient.

### Wg reporter assay

The Wg reporter assay was carried out by conventional methods. WISIR vector that contains both firefly luciferase under the control of a Wg-responsive promoter and *Renilla* luciferase under the control of a Copia promoter was transfected into S2R+ cells. After one day of culture, cells were splitted to a 48 well plate and incubated for 3~4 h until the experimental treatment started. After 24 h of treatment, cells were lysed by following the manufacturer’s instructions of the Dual-Luciferase Repoter Assay System (Promega). Each condition was tested in triplicate.

## Supplementary information

Supplemental figure legends

Supplementary Figure 1

Supplementary Figure 2

Supplementary Figure 3

Supplementary Figure 4

Supplementary Figure 5

Supplementary Figure 6

Supplementary Figure 7

Supplementary Figure 8

Supplementary Figure 9

Supplementary Figure 10
