# Supplementary material for: ADAMTS Sol narae cleaves extracellular Wingless to generate a novel active form that regulates cell proliferation in Drosophila
Source: Cell Death Dis. 2019 Jul 22;10(8):564. doi: 10.1038/s41419-019-1794-8 (PMC6646336; doi:10.1038/s41419-019-1794-8)
Supplement: Supplementary file 1 — Supplemental figure legends [file 41419_2019_1794_MOESM1_ESM.docx]

**Figure S1. Sona is secreted by exosomal transport**

(a) A P100 fraction from *S2 GFP-wg* cells was analyzed by NTA. The average diameter is about 170 nm. (b) A sucrose step gradient of P100 fractions obtained from S2 *sona-HA* or S2 *GFP-wg* cell culture. The active Sona-HA and GFP-Wg were detected with anti-HA and anti-GFP, respectively. Syntaxin 1A was present in similar fractions. (c, d) Immuno-EM of P100 fractions from S2 cells transfected with no DNA as a negative control (c) and transfected with *sona-HA* cDNA (d). Anti-HA antibody and gold-conjugated secondary antibody were used. (e, f) Vesicles expressing both CD63-GFP and Sona-HA in the wing disc of *nub*>*CD63-GFP*, *sona-HA, Gal80^ts^* larvae. These two proteins were transiently expressed for only three hours by *nubbin-Gal4* using *Gal80^ts^* system ^1, 2^ in order to avoid lethality induced by overexpression of CD63-GFP. Vesicles containing CD63 and Sona are marked with arrows in the DV midline (e). Vesicles containing CD63, endogenous Wg and Sona-HA are marked with arrows (f). Scale bar, 7.5 μm.

**Figure S2. Sona does not co-localize with lysosomal vesicles**

Because Sona and Wg are co-localized in Rab5-positive early endosomal vesicles ^3^ that are delivered to either the lysosomal degradation pathway or to the exosomal secretory pathway ^4, 5^, we asked whether Sona is co-localized with the lysosomal marker. The Sona-Pro antibody was used to recognize endogenous Sona in (a) and (c), and the anti-HA antibody was used to detect ectopic Sona-HA in (b). (a) Rab7^+^ vesicles are often juxtaposed with Sona^+^ vesicles (arrows) but they rarely overlapped in *wg*>*rab7-YFP* wing discs (8.95±0.95 %, n=1007), (b, c) There is no co-localization of Sona and HRS (5.37±1.51 %, n=1675) in *nub*>*sona-HA* wing discs (b) or Sona and LAMP (6.19±0.20 %, n=501) in *ap>GFP-lamp* wing discs (c). Scale bar, 7.5 μm.

**Figure S3. Sona is a specific protease for Wg cleavage**

(a, b) Western blots of CX and P100 obtained from S2 *GFP-wg* cells transfected with *sonaE475A* (a, b) or *sona* (a’, b’). Red arrows indicate GFP-Wg^FL^, and the red arrowhead indicates the 23 kDa Wg-CTD^L1^ fragment after long exposure. The 65 and 60 kDa GFP-Wg fragments are indicated by a black arrow and a black arrowhead, respectively (b’). (c) The effect of EDTA on Wg cleavage by Sona. Western blots of CX (c), SN_Δ_ (c’), and P100 (c’’) fractions obtained from S2 *GFP-wg* cells transfected with *sona* and cultured in medium containing 0–2.1 nmol EDTA. Black arrows indicate the 65 kDa GFP-Wg-NTD^L2^ fragments. (d-f) *In vitro* assay for cleavage of heterologous combination between extracellular Sona and Wg. The P100 fraction from S2 *GFP-wg* cell culture and that from S2 *sona-HA* cell culture (or control S2 *HA* cell culture) were mixed and incubated. Then, the mixture was centrifuged to separate the P100 fraction and the SN_Δ_ fraction for western analysis in (d). The P100 fraction from S2 *GFP-wg* cell culture and the SN_Δ_ fraction from S2 *sona-HA* cell culture (or control S2 *HA* cell culture) were mixed and incubated. Then, the P100 fraction was obtained from this mixture for western analysis in (e). Black arrows indicate the 65 kDa Wg-NTD^L2^ fragment in (d, e). Cleaved Wg products were detected only in the P100 but not in the SN_Δ_ fraction, demonstrating that cleaved Wg fragments stayed on exosomes in (d). The SN_Δ_ fraction from S2 *GFP-wg* cell culture and the P100 fraction from S2 *sona-HA* cell culture (or control S2 *HA* cell culture) were mixed and incubated. Then, the SN_Δ_ fraction was obtained from this mixture for western analysis in (f). Black arrow and red arrowhead indicate the 65 kDa Wg-NTD^L2^ fragment and 23 kDa Wg-CTD^L1^ fragment in (f), respectively.

**Figure S4. Wg is a substrate of Sona**

(a) The procedure of *in vitro* assay using GST-linker-CTD protein synthesized in *E.coli*. The detailed procedure is described in Materials and Methods. Since Sona expressed in *E. coli* was not functional due to the lack of glycosylation (data not shown), purified GST-linker-CTD was incubated with active Sona-HA immunoprecipitated from the SN_Δ_ fraction of *sona-HA* S2 cell culture. (b, c) Western blots of GST-linker-CTD incubation mixtures with or without Sona by 4D4 in (b) and anti-HA in (c) antibodies. ‘Media’ lane contains GST-linker-CTD protein incubated with M3 media, and HA lane contains the immunoprecipitated proteins with anti-HA antibody from the SN_Δ_ fraction of S2 cell culture. Red arrow and red arrowhead indicate full-length GST-linker-CTD and cleaved 17 kDa CTD^L1^ fragment, respectively in (b). The pound signs (#) mark degraded products of the GST-linker-CTD independent of the active Sona in (b). A Black arrow indicates active Sona from the S2 *sona-HA* cell culture immunoprecipitated using anti-HA antibody in both CX and SN_Δ_ fractions, a red arrow marks full-length Sona, and two asterisks indicate heavy and right chains of Rat IgG in (c). GST-linker-CTD was not cleaved by the immunoprecipitated active Sona in CX, indicating that only extracellular Sona is active in Wg cleavage (Fig. 1f and Supplemental Fig. 3A, B). (d) CX and P100 fractions were obtained from the cultured media of S2 cells expressing both Wg and Sona-HA, and were treated with Peptide-N-glycosidase F (PNGase) to remove glycosyl residues. The position of the Wg-CTD^L1^ fragment is shifted down by PNGase from 23 kDa (a black arrowhead) to 17 kDa (a red arrowhead) in P100 fraction.

**Figure S5. Cleaved products of GFP-Wg *in vivo***

(a) A diagram of GFP-Wg encoded by *UAS-GFP-wg* transgene. Red, green and yellow bars represent full-length and cleaved Wg fragments as indicated. Grey bars indicate the undetectable Wg-CTD^L2^ fragment. (b) Extracellular patterns of GFP signal and the 4D4 in the basal ECM of *wg*>*GFP-wg* wing discs*.* The regions marked with squares in (b) is magnified in (c). Wg-producing regions are marked by brackets in (c). Representative Green, red and yellow signals are marked with an arrowhead, an arrow, and a circle, respectively. Scale bar, (b), 40 μm; (c), 5 μm.

**Figure S6. Wg-CTD is not active in the luciferase assay with S2 R+ cells**

Artificially engineered Wnt7a-CTD (Wnt7a-CT) induces Wnt luciferase activity using Topflash constructs ^6^. The *wg-CTD* construct was generated based on the corresponding region in Wnt7a-CTD and used in Drosophila S2 R+ cell culture system. (a) Wg-CTD reporter assay using TopFlash construct in S2 R+ cells. Full-length Wg and GFP-Wg expressed by actin promoter have a strong luciferase activity in (a) as a positive control. In contrast, Expression of Wg-CTD and Wg-NTD alone (a) did not induce any luciferase activity. (b) Wg reporter assay with coexpressed Sona using TopFlash construct in S2 R+ cells. When the fixed amount of the conditioned medium from S2 *wg* cell culture was mixed with the increasing amounts of the conditioned medium from S2 *sona-HA* cell culture, the luciferase activity negatively correlated with amount of the Sona-HA. This suggests that the amount of full-length Wg is reduced by Sona. The results obtained from Wg-CTD luciferase assay in S2 R+ cells did not reflect the *in vivo* results.

**Figure S7. Generation of Wg-NTD and Wg-CTD transgenic flies and their phenotypes**

(a) Construction strategy to generate *UAS-wg-NTD, UAS-wg-CTD, UAS-GFP-wg-NTD* and *UAS-wg-mycCTD* transgenic flies. The Myc tag was inserted just upstream of the CTD to be located between the L2 and the CTD. (b-f) Wg-NTD and Wg-CTD expression by *nub-Gal4*. *nub>GFP-wg-NTD* wing (b) and *nub>wg-NTD* (no tag) wing (e) did not induce any visible phenotypes, but both *nub>wg-mycCTD* wing (c) and *nub>wg-CTD* wing (f) were smaller and cross-veins disappeared. (g, h) GFP signal is absent in the control *nub-Gal4* (g), and GFP signal from GFP-Wg-NTD expressed in *nub>GFP-wg-NTD* wing discs. (i, j) Staining by WgN (N-terminal epitope antibody) of *nub-Gal4* (i) and *nub>wg-NTD* (j) wing discs. (k-o) Overexpression of Wg-CTD results in size reduction of wings and eyes. The posterior regions of the *en>GFP, wg-mycCTD* wing disc (l) and the *en>GFP, wg-CTD* wing disc (m) are much smaller than that of the *en>GFP* control disc (k). *ci>wg-mycCTD* flies have small and rough eyes (o) compared to the control *CS* (n). Scale bar, 60 μm.

**Figure S8. Wg-mycCTD expression rescues *sona RNAi* phenotypes**

(a, b) Flies incubated at 30°C for the experiment presented in Fig. 4s. (c, d) Wg-mycCTD expression partially suppresses the lethality induced by *sona RNAi*. All *ptc*>*sonaRNAi* flies are early larval lethal (c). 28.1% of *ptc*>*sonaRNAi,* *wg-mycCTD* flies are late pupal lethal and 7.5% of them are pupal lethal (n =160 each) (c, d). (e-g) The *ptc>GFP, wg-mycCTD* wing in (f) shows loss of the wing margin cell (arrow) and anterior cross-vein (ACV) (arrowhead). *ptc>GFP, wg-mycCTD, sona RNAi* wings lack ACV at a lower frequency with normal margin (g).

**Figure S9. Wg-CTD expression increases the level of Dll and Vg, but not Sens**

The flies were shifted from 18°C to 30°C for 10 hours during the late second and the early first larval instar for transient expression from (f) to (k). (a, b) The level of Dll is not changed in *en>GFP, wg-NTD* discs (a). No tagged Wg-CTD expression also increased the level of Dll in the posterior region of the *en>GFP, wg-CTD* discs (b). (c-e) Wg-CTD alone may be unable to carry out the full functionality of Wg signaling. *wg^CX4^ UAS-GFP-wg /CyO-GFP* or *wg^CX4^ UAS-wg-mycCTD /CyO-GFP* males were crossed with *wg^Gal4^/CyO-GFP* virgins flies in (c, d) to check whether the phenotype of *wg^Gal4^/ wg^CX4^* can be rescued by Wg-CTD expression. Because *wg^Gal4^* is one of *wg* mutant allele caused by *Gal4* P-element insertion, *wg^Gal4^ / wg^CX4^* was embryonic lethal as reported ^7^. While eight *wg^CX4^ UAS-GFP-wg/ wg^Gal4^* ­adults in (e) and three dead pupae (n=65) were obtained, neither adults nor dead pupae were found in *wg^CX4^ UAS-wg-mycCTD/ wg^Gal4^* flies (n=70). (f, g) Cyc D expression in *en>GFP, Gal80^ts^* (f) and *en>GFP, Gal80^ts^, wg-mycCTD* (g) wing discs. The arrow in (g) indicates the posterior region where Cyc D is increased. (h-g) Expression pattern of Sens was identical in control (h), *en>GFP, Gal80^ts^, wg-mycCTD* (i), and *ptc>GFP-wg-NTD* wing discs (j). (k, l) Transient expression of GFP-Wg-NTD (k) and prolonged expression of Wg-mycCTD (l) induced no ectopic bristles.

**Figure S10. Sona generates Wg-CTD, a novel active Wg form**

A model to show the generation of Wg-CTD by Sona and comparison between the function of full-length Wg and Wg-CTD. (1) When Sona is absent, full-length Wg is stable in extracellular space. This full-length Wg induces Wg signaling such as Arm, Vg, Dll and Sens by binding to Fz receptor. (2) When Sona is present, Wg is cleaved to Wg-CTD and Wg-NTD. Although Wg-CTD is unstable, it can induce Arm, Vg, Dll except Sens. Furthermore, Wg-CTD is also able to increase the level of CycD. In case of Wg-NTD, it does not have activity for Wg signaling.

1. Laulagnier, K., Vincent-Schneider, H., Hamdi, S., Subra, C., Lankar, D., Record, M. Characterization of exosome subpopulations from RBL-2H3 cells using fluorescent lipids. *Blood Cells Mol Dis*. **35**, 116-121 (2005).

2. Escola, J. M., Kleijmeer, M. J., Stoorvogel, W., Griffith, J. M., Yoshie, O., Geuze, H. J. Selective enrichment of tetraspan proteins on the internal vesicles of multivesicular endosomes and on exosomes secreted by human B-lymphocytes. *J Biol Chem*. **273**, 20121-20127 (1998).

3. Kim, G.W. & Won, J. H. *et al.* Sol narae (Sona) is a Drosophila ADAMTS involved in Wg signaling. *Sci Rep-Uk* **6**. (2016)

4. Marois, E., Mahmoud, A., Eaton, S. The endocytic pathway and formation of the Wingless morphogen gradient. *Development* **133**, 307-317 (2006).

5. Mathivanan, S., Ji, H., Simpson, R. J. Exosomes: extracellular organelles important in intercellular communication. *J Proteomics* **73**, 1907-1920 (2010).

6. von Maltzahn, J., Zinoviev, R., Chang, N. C., Bentzinger, C. F., Rudnicki, M. A. A truncated Wnt7a retains full biological activity in skeletal muscle. *Nat Commun.* **4**, 2869 (2013).

7. Giraldez, A. J., Copley, R. R., Cohen, S. M. HSPG modification by the secreted enzyme Notum shapes the Wingless morphogen gradient. *Dev Cell*. **2**, 667-676 (2002).
