# Supplementary figures and images for: ADAMTS Sol narae cleaves extracellular Wingless to generate a novel active form that regulates cell proliferation in Drosophila
Source: Cell Death Dis. 2019 Jul 22;10(8):564. doi: 10.1038/s41419-019-1794-8 (PMC6646336; doi:10.1038/s41419-019-1794-8)

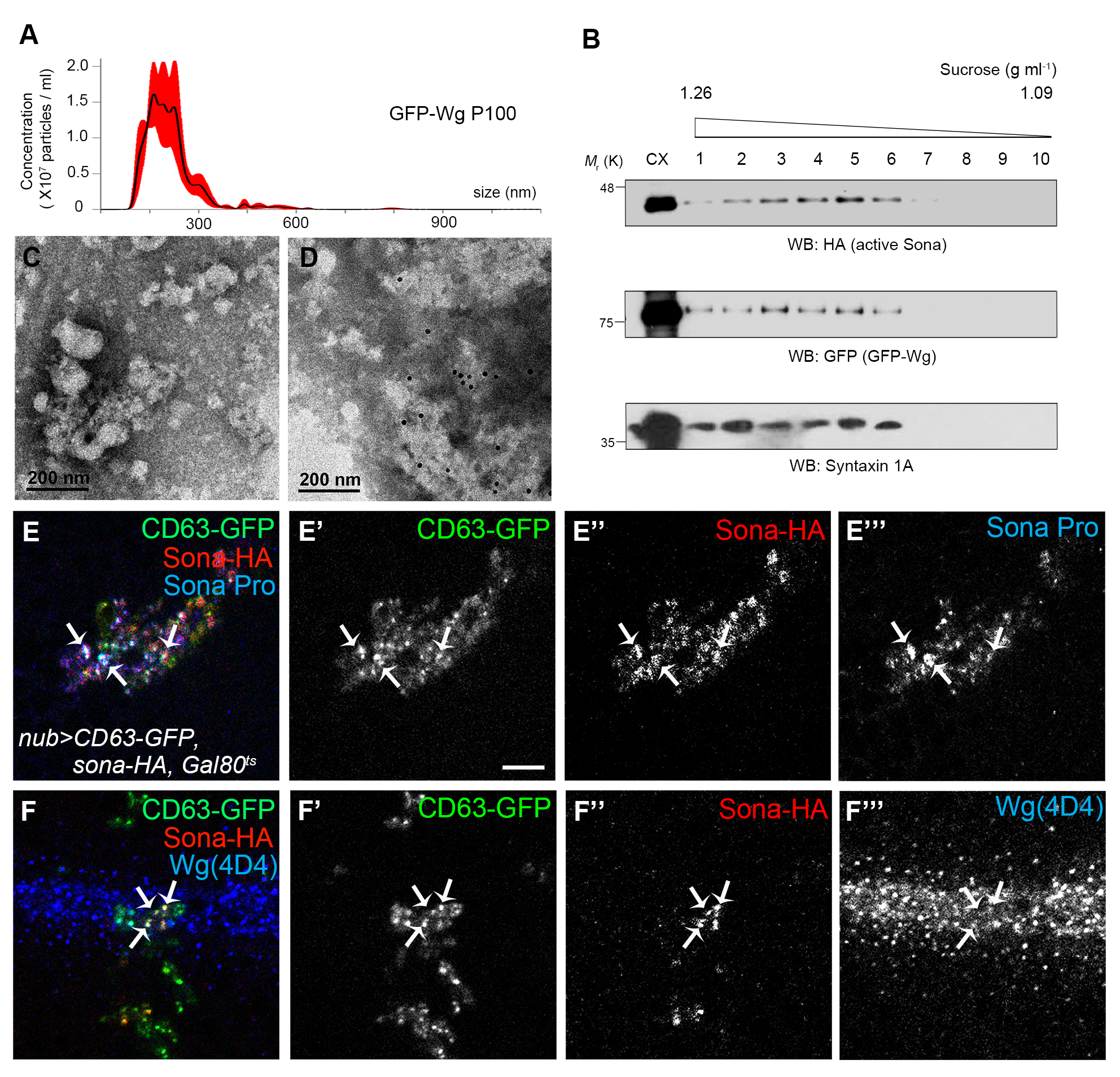

Supplement: Supplementary file 2 — Supplementary Figure 1 [file 41419_2019_1794_MOESM2_ESM.tif]

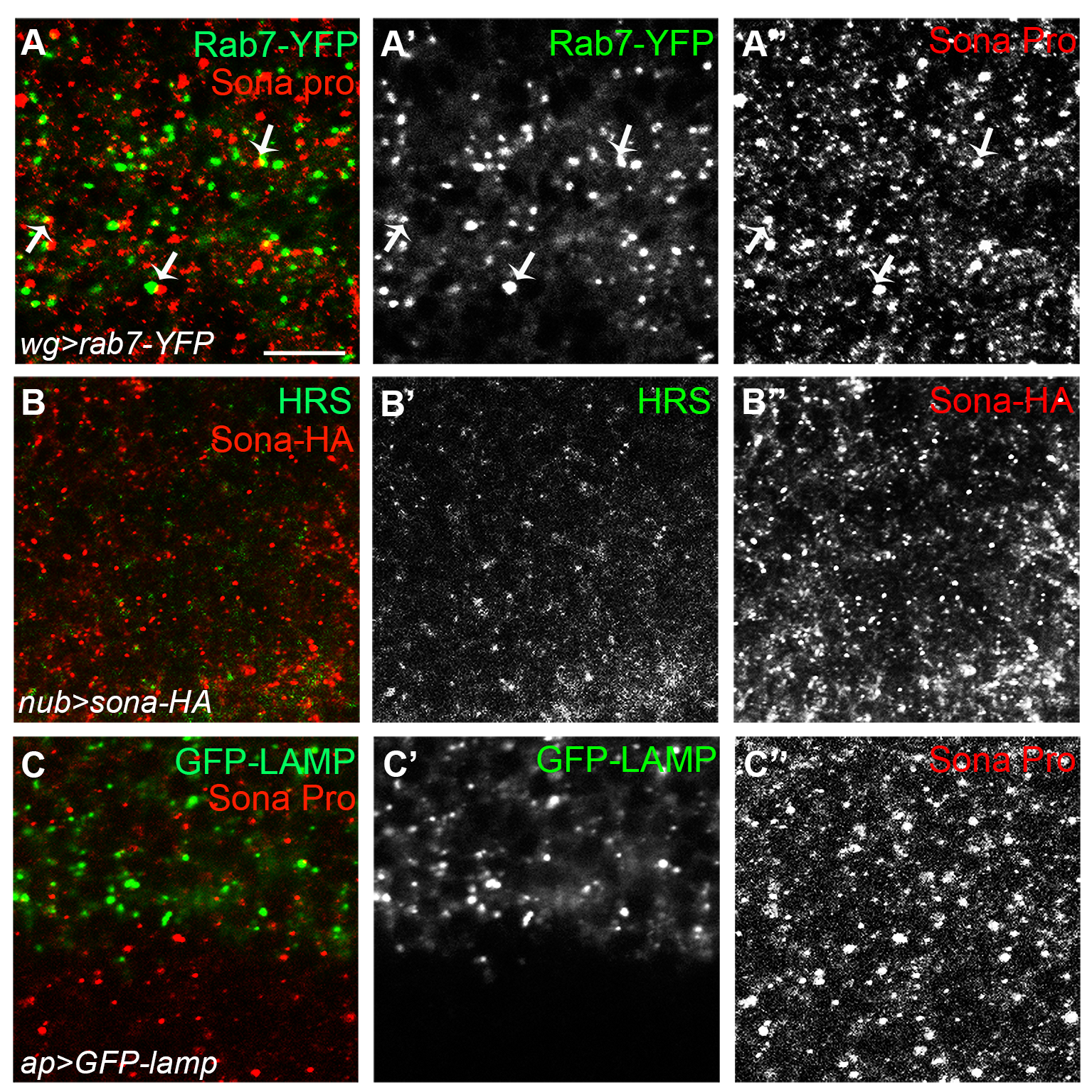

Supplement: Supplementary file 3 — Supplementary Figure 2 [file 41419_2019_1794_MOESM3_ESM.tif]

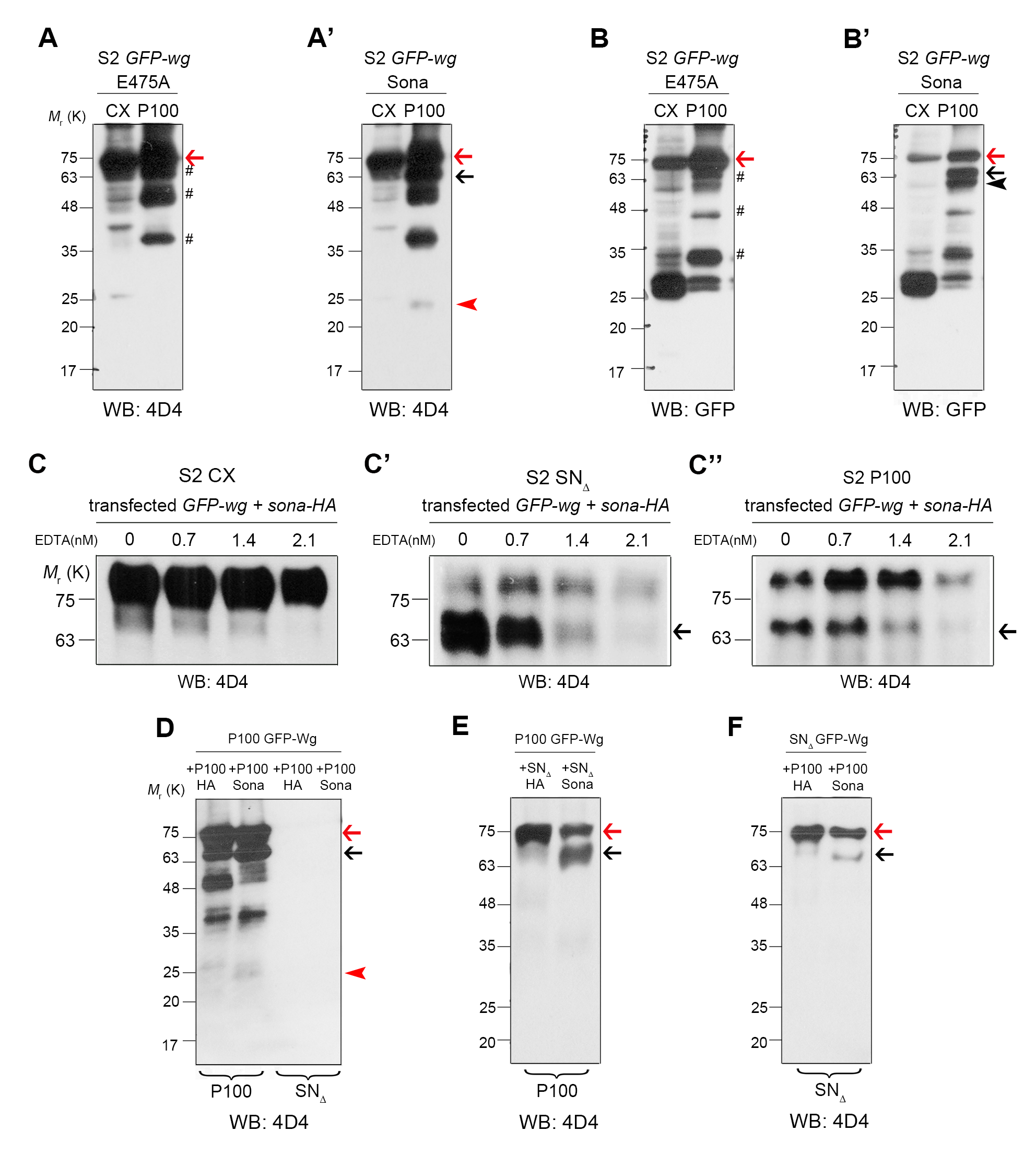

Supplement: Supplementary file 4 — Supplementary Figure 3 [file 41419_2019_1794_MOESM4_ESM.tif]

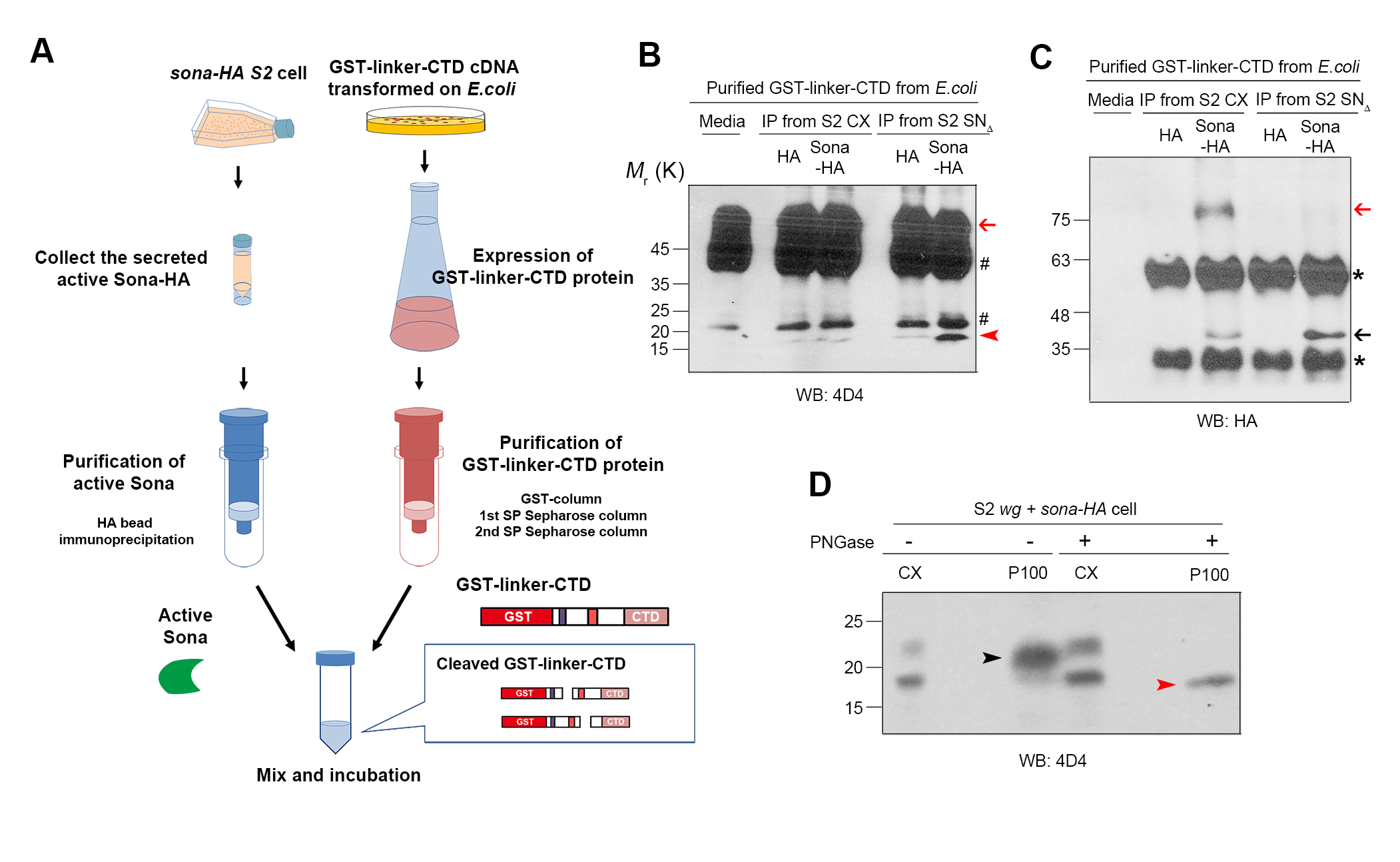

Supplement: Supplementary file 5 — Supplementary Figure 4 [file 41419_2019_1794_MOESM5_ESM.tif]

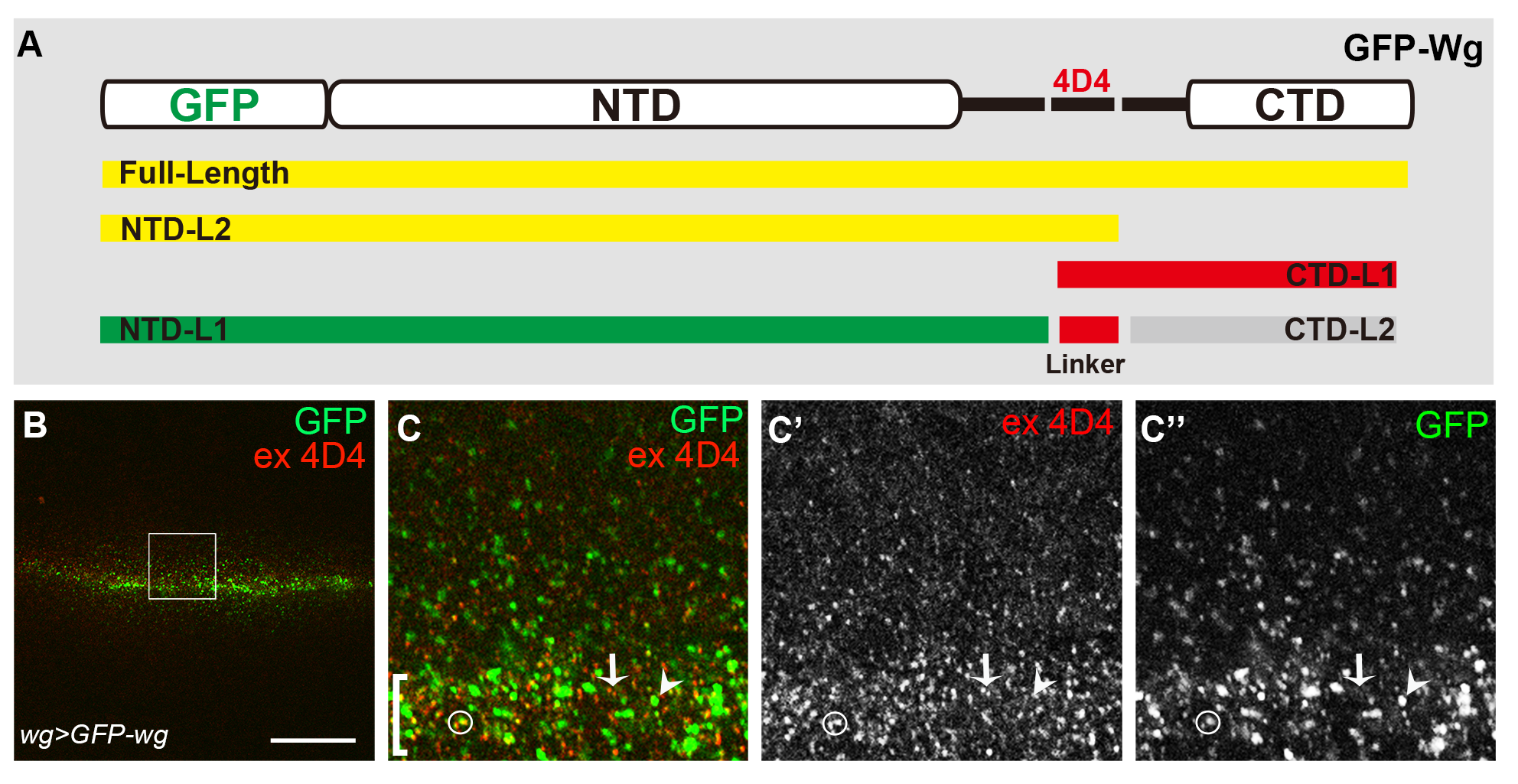

Supplement: Supplementary file 6 — Supplementary Figure 5 [file 41419_2019_1794_MOESM6_ESM.tif]

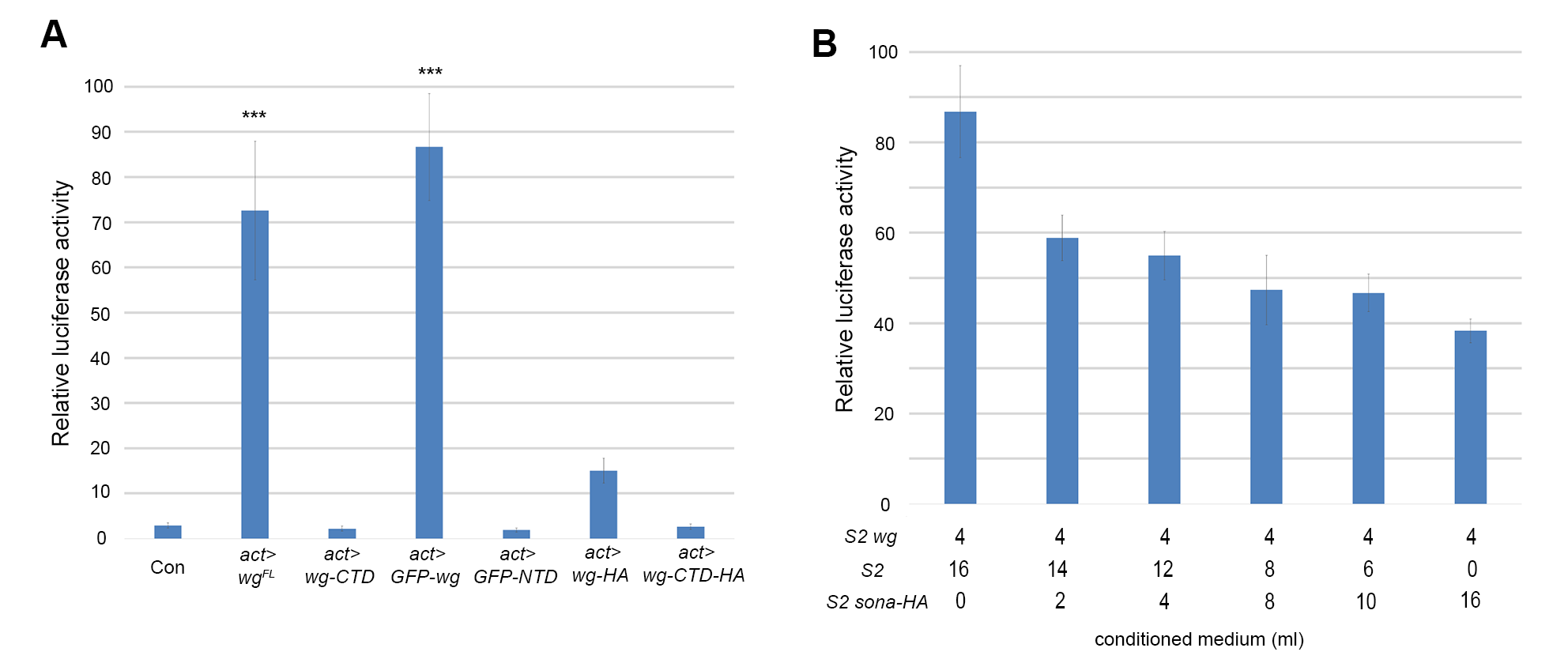

Supplement: Supplementary file 7 — Supplementary Figure 6 [file 41419_2019_1794_MOESM7_ESM.tif]

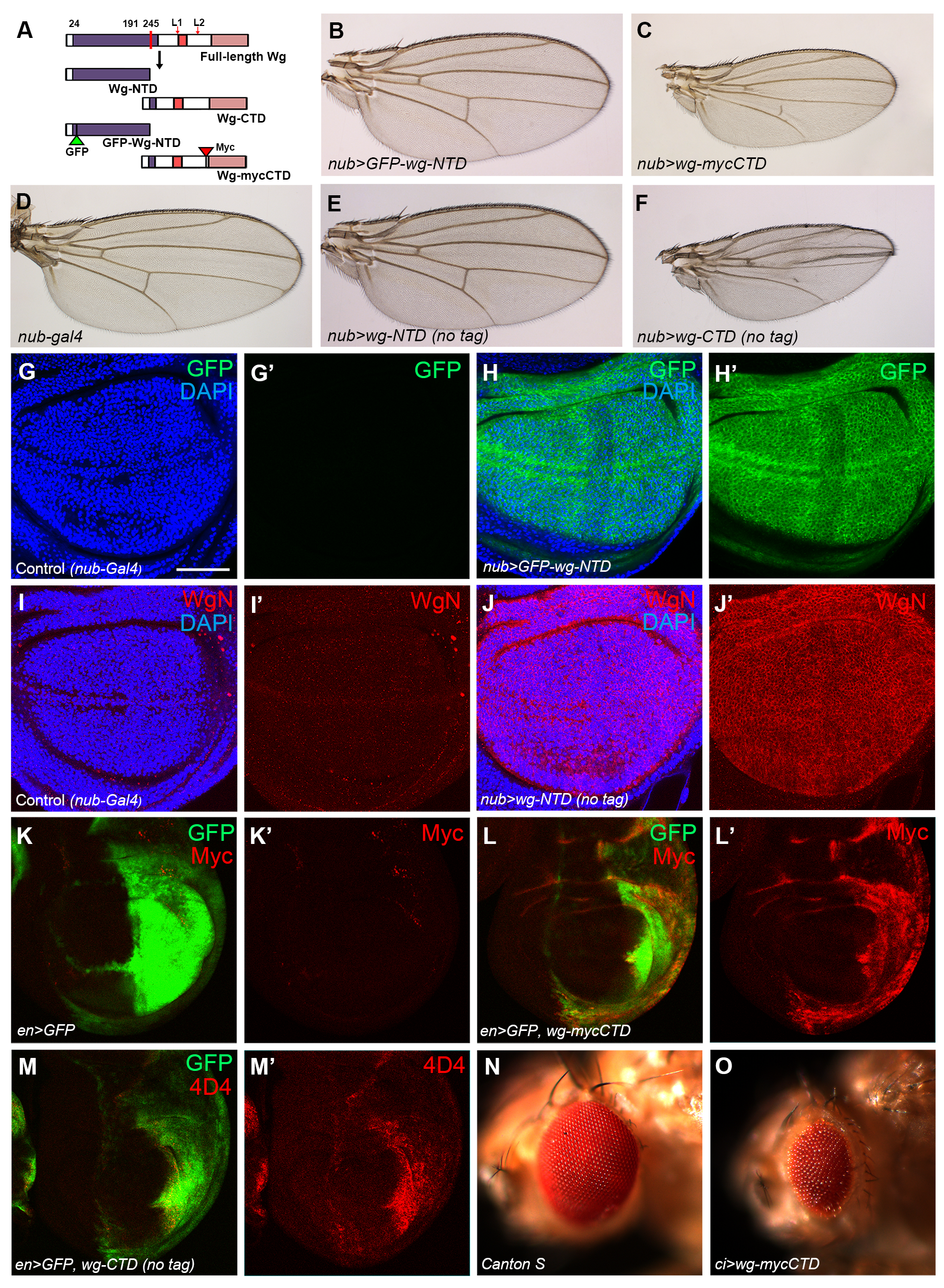

Supplement: Supplementary file 8 — Supplementary Figure 7 [file 41419_2019_1794_MOESM8_ESM.tif]

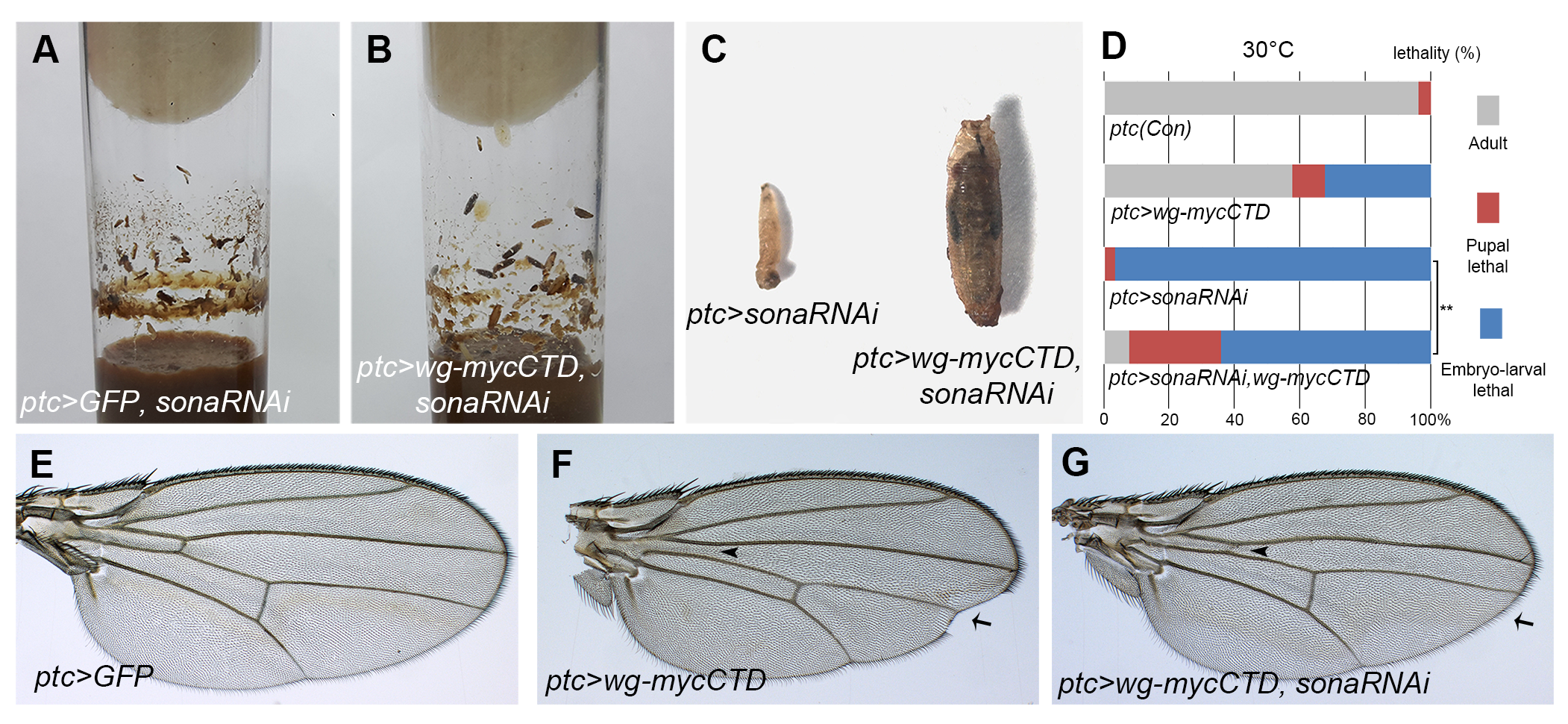

Supplement: Supplementary file 9 — Supplementary Figure 8 [file 41419_2019_1794_MOESM9_ESM.tif]

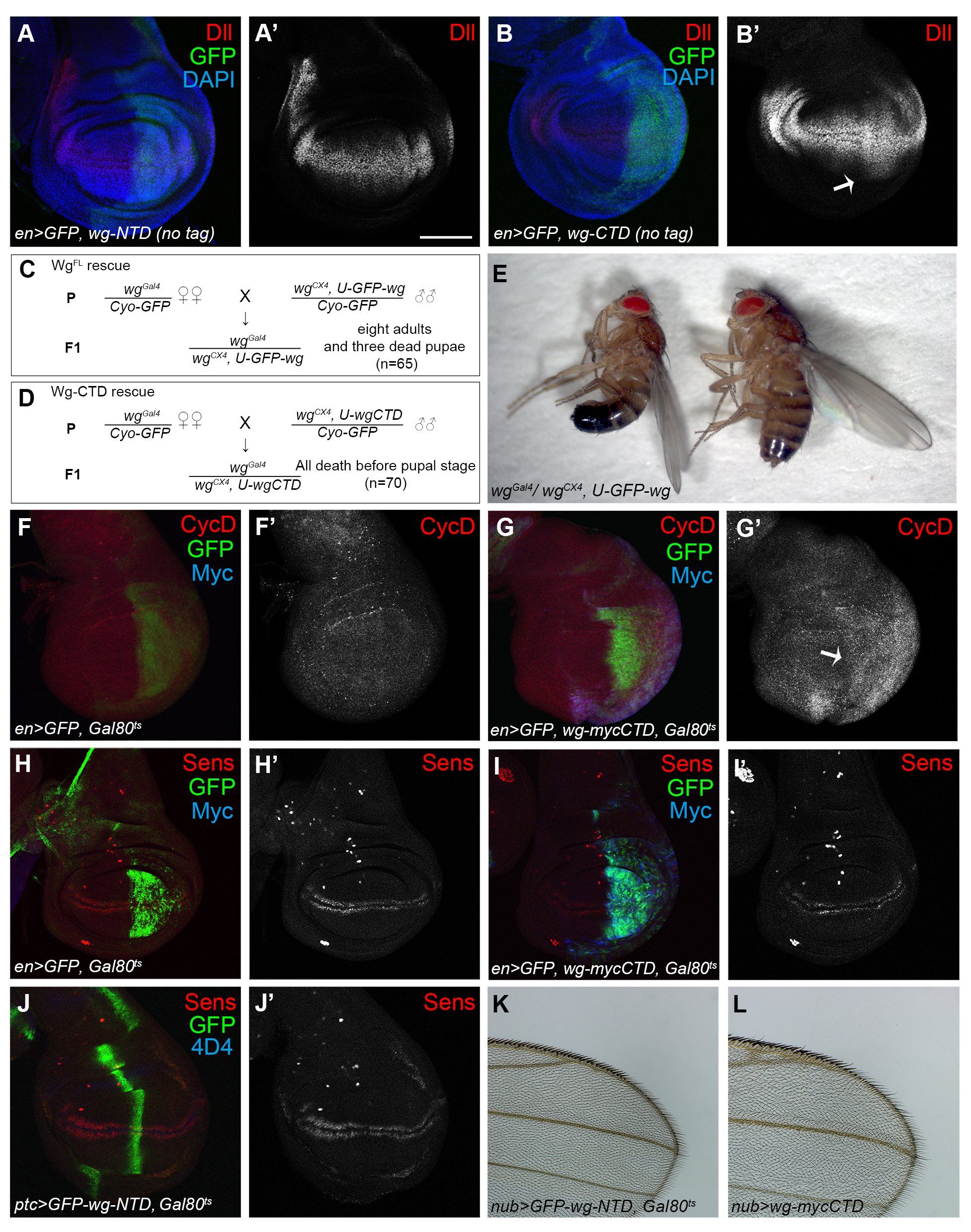

Supplement: Supplementary file 10 — Supplementary Figure 9 [file 41419_2019_1794_MOESM10_ESM.tif]

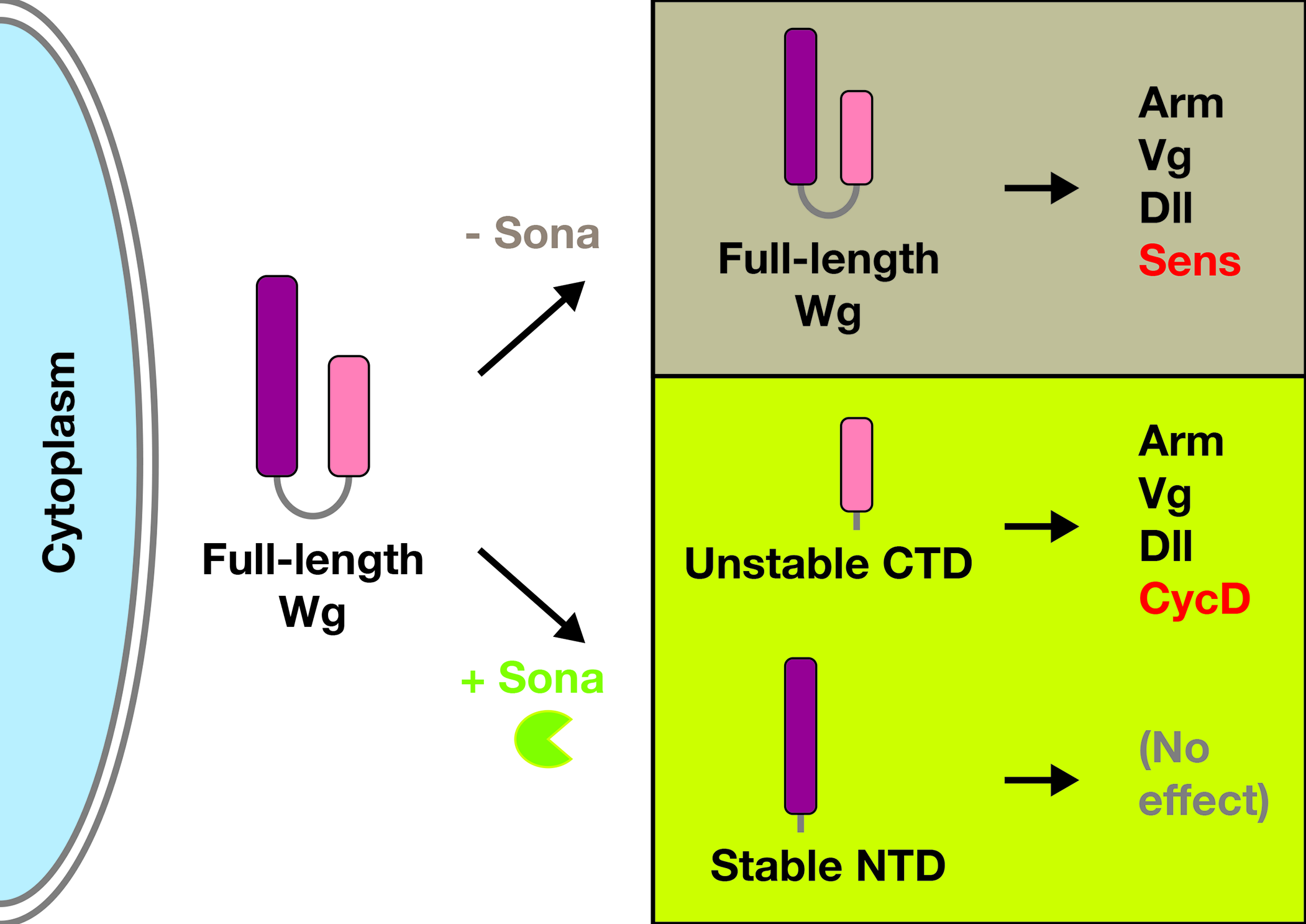

Supplement: Supplementary file 11 — Supplementary Figure 10 [file 41419_2019_1794_MOESM11_ESM.tif]
